# *Bacillus cereus* non-haemolytic enterotoxin activates the NLRP3 inflammasome

**DOI:** 10.1038/s41467-020-14534-3

**Published:** 2020-02-06

**Authors:** Daniel Fox, Anukriti Mathur, Yansong Xue, Yunqi Liu, Wei Hong Tan, Shouya Feng, Abhimanu Pandey, Chinh Ngo, Jenni A. Hayward, Ines I. Atmosukarto, Jason D. Price, Matthew D. Johnson, Nadja Jessberger, Avril A. B. Robertson, Gaetan Burgio, David C. Tscharke, Edward M. Fox, Denisse L. Leyton, Nadeem O. Kaakoush, Erwin Märtlbauer, Stephen H. Leppla, Si Ming Man

**Affiliations:** 1 Department of Immunology and Infectious Disease, The John Curtin School of Medical Research, The Australian National University, Canberra, Australia; 2 Lipotek Pty Ltd. The John Curtin School of Medical Research, The Australian National University, Canberra, Australia; 3 Research School of Biology, The Australian National University, Canberra, Australia; 4 Department of Veterinary Sciences, Faculty of Veterinary Medicine, Ludwig-Maximilians-Universität München, Oberschleißheim, Germany; 5 School of Chemistry and Molecular Biosciences, The University of Queensland, Brisbane, QLD 4072 Australia; 6 Department of Applied Sciences, Northumbria University, Newcastle Upon Tyne, UK; 7 Medical School, The Australian National University, Canberra, Australia; 8 School of Medical Sciences, UNSW Sydney, Sydney, NSW 2052 Australia; 9 Microbial Pathogenesis Section, Laboratory of Parasitic Diseases, National Institute of Allergy and Infectious Diseases, National Institutes of Health, Bethesda, MD 20892 USA

**Keywords:** Pattern recognition receptors, Pathogens

## Abstract

Inflammasomes are important for host defence against pathogens and homeostasis with commensal microbes. Here, we show non-haemolytic enterotoxin (NHE) from the neglected human foodborne pathogen *Bacillus cereus* is an activator of the NLRP3 inflammasome and pyroptosis. NHE is a non-redundant toxin to haemolysin BL (HBL) despite having a similar mechanism of action. Via a putative transmembrane region, subunit C of NHE initiates binding to the plasma membrane, leading to the recruitment of subunit B and subunit A, thus forming a tripartite lytic pore that is permissive to efflux of potassium. NHE mediates killing of cells from multiple lineages and hosts, highlighting a versatile functional repertoire in different host species. These data indicate that NHE and HBL operate synergistically to induce inflammation and show that multiple virulence factors from the same pathogen with conserved function and mechanism of action can be exploited for sensing by a single inflammasome.

## Abstract (web summary)

The *Bacillus* haemolytic enterotoxin haemolysin BL has been shown to activate the NLRP3 inflammasome. Here the authors show that a non-haemolytic enterotoxin (NHE) from *B. cereus* can also activate the NLRP3 inflammasome with a similar mechanism of lytic pore formation.

## Introduction

*Bacillus cereus* is a clinically important human foodborne pathogen. This Gram-positive and rod-shaped bacterium is found ubiquitously in the environment and in undercooked and processed food products^1^. Ingestion of *B. cereus* endospores often leads to germination and propagation of viable vegetative cells in the human gastrointestinal tract, which may lead to emetic and diarrheal syndromes largely depending on the production of enterotoxins^2^. Of concern is the potential for *B. cereus* to cause often-fatal extra-gastrointestinal disease in immune-compromised patients, including systemic bacterial septicemia, ocular infections, anthrax-like pneumonia, cutaneous gas-gangrene-like infections, and infections of the central nervous system^1^.

The critical components of host innate immune defence against invading pathogens are cytosolic inflammasome complexes^3–5^. Several inflammasome sensor proteins have been identified, including AIM2, NAIP-NLRC4, NLRP1, NLRP3, NLRP6, NLRP9b, Pyrin, and caspase-11^6,7^. These inflammasome sensors, when activated, can recruit the inflammasome adaptor protein apoptosis-associated speck-like protein containing a caspase activation and recruitment domain (ASC, also known as PYCARD), which further recruits the cysteine protease caspase-1^8,9^. Caspase-1 is required to induce cleavage of the proinflammatory cytokines pro-interleukin-1β (pro-IL-1β) and pro-IL-18, as well as the pro-pyroptotic factor, gasdermin D (GSDMD)^10–13^, to drive an inflammatory form of programmed cell death known as pyroptosis.

Inflammasome sensor proteins can recognize pathogen-associated molecular patterns (PAMPs), danger-associated molecular patterns, and the more recently proposed homeostasis-altering molecular processes^14,15^.

Bacterial toxins are key virulence factors that represent a class of PAMPs, which are potent activators of inflammasome sensors, leading to inflammation and cell death in the host^14,16^. The lethal factor from the anthrax-causing pathogen *Bacillus anthracis* can enter the host cell cytoplasm and induce cleavage of the inflammasome sensor NLRP1b^17–23^. Unlike the lethal factor, the toxins TcdA and TcdB produced by the gastrointestinal pathogen *Clostridium difficile* inactivate host Rho-GTPases in the cytoplasm. This homeostasis-altering event induces dephosphorylation and activation of the inflammasome sensor Pyrin, triggering assembly of the Pyrin inflammasome^24^. We and others have shown that more structurally diverse toxins can induce activation of the NLRP3 inflammasome via a mechanism independent of entry to the host cell cytoplasm. These toxins include haemolysins of *Staphylococcus aureus*^25–28^ and *Escherichia coli*^29,30^, and haemolysin BL (HBL) of *B. cereus*^31^.

Of particular interest is that *B. cereus* isolates that lack HBL can cause inflammation and disease in humans^32–34^, suggesting that other non-redundant virulence factors are critical in the pathogenesis of this pathogen. Here, we identify that non-haemolytic enterotoxin (NHE) of *B. cereus* is able to induce activation of the NLRP3 inflammasome and pyroptosis via a mechanism targeting the plasma membrane of host cells. We also demonstrate that NHE subunits assemble to form a functional pore, driving efflux of cytosolic potassium. This toxin kills cell types from multiple lineages and host origin, highlighting its functional repertoire in different host species. Our results reveal that multiple functionally conserved toxins from *B. cereus* are targeted by a single inflammasome to initiate inflammation and cell death in the host. This host strategy offers a single pathogen sensor the flexibility to mediate the recognition of functionally conserved toxins, often produced by phylogenetically diverse bacterial species or even within different strains of a single bacterial species.

## Results

### A non-redundant secreted factor of *B. cereus* activates NLRP3

**Fig. 1:**
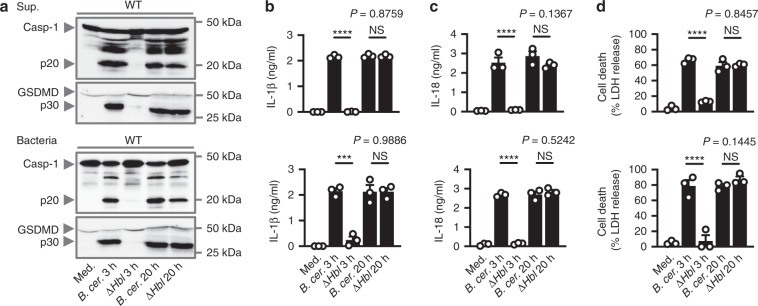
A secreted factor of *B. cereus* activates the inflammasome independently of HBL. **a** Immunoblot analysis of pro-caspase-1 (Casp-1), the active caspase-1 p20 subunit, pro-pyroptotic effector protein gasdermin D (GSDMD), the active GSDMD p30 subunit of WT BMDMs left untreated [Medium alone (Med.)] or LPS-primed and assessed either 3 h or 20 h after stimulation with the supernatant of WT *B. cereus* (*B. cer*.) or Δ*Hbl B. cereus* (Δ*Hbl*) (top; Supernatant (Sup.)) or infection with *B. cer*. or Δ*Hbl* (bottom; m.o.i. of 5). Release of IL-1β **b**, IL-18 **c**, and cell death **d** of BMDMs as treated in **a**. NS, not significant, ***P < 0.001 and ****P < 0.0001 (student’s unpaired *t* test **b**–**d**). Each symbol represents an independent experiment **b**–**d**. Data are representative of three independent experiments (*n* = 3, **a**–**d**; mean and s.e.m. in **b**–**d**). Source data are provided as a Source Data file.

We have previously demonstrated that innate immune recognition of *Bacillus cereus* infection requires inflammasome-mediated sensing of a toxin known as HBL^31^. Stimulation of primary wild-type (WT) bone marrow-derived macrophages (BMDMs) with the supernatant of WT *B. cereus* led to activation of caspase-1, cleavage of GSDMD, secretion of IL-1β and IL-18, and pyroptosis within 3 h, whereas stimulation of WT BMDMs with the supernatant of an isogenic mutant of *B. cereus* lacking HBL (Δ*Hbl B. cereus*) did not (Fig. 1a–d), consistent with our previous findings^31^. However, we were surprised to find that WT BMDMs treated overnight with the supernatant of Δ*Hbl B. cereus* underwent robust activation of caspase-1, cleavage of GSDMD, secretion of IL-1β and IL-18, and induction of cell death (Fig. 1a–d). We further confirmed that live Δ*Hbl B. cereus* bacteria also induced activation of the inflammasome in WT BMDMs following overnight stimulation (Fig. 1a–d). These findings suggest that an additional non-redundant secreted factor of *B. cereus* might be sensed by the inflammasome.

**Fig. 2:**
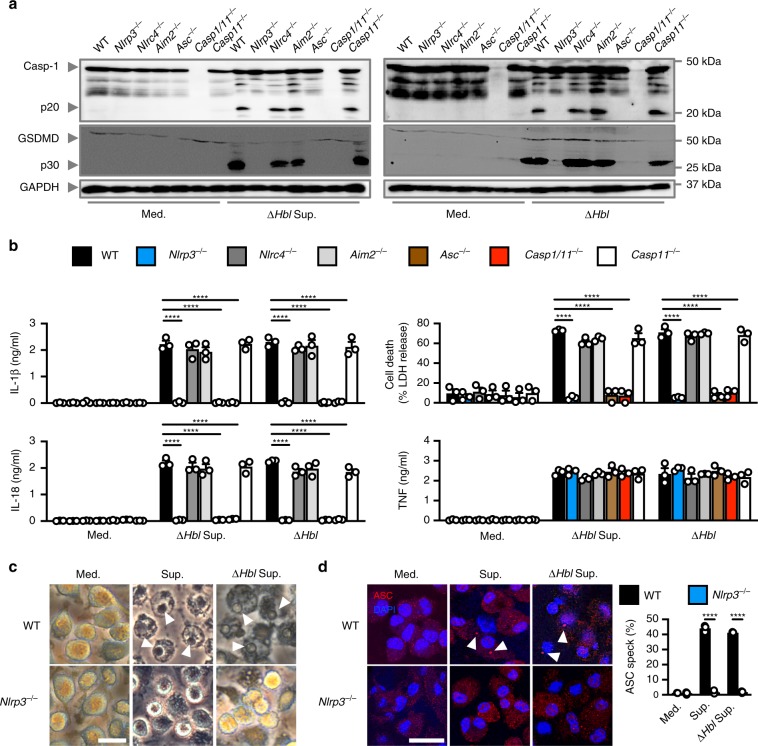
The secreted factor of *B. cereus* activates the NLRP3 inflammasome. **a**, Immunoblot analysis of caspase-1, gasdermin D, and GAPDH (loading control) in WT or mutant BMDMs left untreated or LPS-primed and assessed 20 h after stimulation with Δ*Hbl* supernatant, or after infection with Δ*Hbl* (m.o.i. of 5). **b** Release of IL-1β, IL-18, and TNF and death of BMDMs after treatment as in **a**. **c** Brightfield microscopy analysis of BMDMs 8 h after stimulation as in **a**. **d** Immunofluorescent analysis of ASC speck formation (red) in BMDMs after treatment as in **a**. Quantification of the prevalence of ASC specks is shown on the right as a percentage of total cells (DAPI). At least 200 cells from each genotype were assessed. Scale bar, 25 μm **c**, **d**. Arrowheads indicate pyroptotic cells **c** or ASC specks **d**. NS, not significant, ****P < 0.0001 (one-way ANOVA with Dunnett’s multiple-comparisons test **b** or student’s unpaired *t* test **d**). Each symbol represents an independent experiment **b**, **d**. Data are representative of three independent experiments (*n* = 3, **a**–**d**; mean and s.e.m. in **b**, **d**). Source data are provided as a Source Data file.

To elucidate the identity of the inflammasome sensor responsible for the recognition of the secreted factor, we stimulated lipopolysaccharide (LPS)-primed primary WT, *Nlrp3*^–/–^, *Nlrc4*^–/–^, *Aim2*^–/–^, *Asc*^–/–^, *Casp1/11*^–/–^, and *Casp11*^–/–^ BMDMs with the supernatant of Δ*Hbl B. cereus*. The supernatant of Δ*Hbl B. cereus* induced caspase-1 activation, cleavage of GSDMD, secretion of IL-1β and IL-18, and cell death in WT, *Nlrc4*^–/–^, *Aim2*^–/–^, and *Casp11*^–/–^ BMDMs (Fig. 2a–c). In contrast, the supernatant of Δ*Hbl B. cereus* did not induce activation of the inflammasome and cell death in *Nlrp3*^–/–^ and *Asc*^–/–^ BMDMs (Fig. 2a–c), indicating that NLRP3 is the cytosolic sensor of the secreted factor of Δ*Hbl B. cereus*. Consistent with the results obtained using cell-free supernatant, we found that live Δ*Hbl B. cereus* bacteria activated the NLRP3 inflammasome (Fig. 2a, b and Supplementary Fig. 1a).

Gene and protein expression analyses of proinflammatory cytokines tumor-necrosis factor (TNF), keratinocyte chemoattractant (KC, also known as CXCL1) and pro-IL-1β, and the phosphorylation status of IκB and ERK between WT and *Nlrp3*^*–*/–^ BMDMs following stimulation by the supernatant or live bacteria of Δ*Hbl B. cereus* were similar (Fig. 2b and Supplementary Fig. 1b–d). These data confirm that the production of inflammasome-independent cytokines was not affected by the genetic deletion of NLRP3, and that the unknown secreted factor did not affect priming of the inflammasome.

We further confirmed that assembly of the inflammasome complex, which can be visualized by immunostaining of the ASC speck^35^, occurred in WT BMDMs treated with the supernatant from either WT *B. cereus* or Δ*Hbl B. cereus* or infected with either WT *B. cereus* or Δ*Hbl B. cereus* (Fig. 2d and Supplementary Fig. 1e). Further, pharmacological inhibition of NLRP3 using the small molecule inhibitor MCC950^36–38^ abrogated caspase-1 activation, secretion of IL-1β and IL-18, and cell death in WT BMDMs stimulated with the supernatant or live bacteria of Δ*Hbl B. cereus* (Supplementary Fig. 1f). Collectively, our data suggest that a putative secreted factor of Δ*Hbl B. cereus* activates the NLRP3 inflammasome.

### The secreted factor is a 30–50 kDa heat-sensitive protein

To decipher the biological identity of the putative secreted inflammasome-activating factor, we stimulated WT BMDMs overnight with the supernatant of Δ*Hbl B. cereus,* which had been heat-treated or treated with proteinase K, DNase, or RNase. We found that the supernatant heated to 50 °C, but not 75 °C and 100 °C, retained its ability to induce cleavage of caspase-1 and GSDMD, secretion of IL-1β and IL-18, and cell death in WT BMDMs (Supplementary Fig. 2a, b). Further, the supernatant treated with proteinase K did not lead to activation of caspase-1, cleavage of GSDMD, secretion of IL-1β and IL-18, and cell death in WT BMDMs, whereas treatment of DNase or RNase did not affect the ability of the supernatant to induce inflammasome responses (Supplementary Fig. 2c, d). To deduce the molecular size of the secreted factor, we used filter-mediated size-fractionation techniques to separate the supernatant of Δ*Hbl B. cereus* into different molecular-sized fractions. We found that the supernatant fractions of >9 kDa, >30 kDa, and <50 kDa, but not fractions of <9 kDa, <30 kDa, and >50 kDa, potentiated activation of the inflammasome (Supplementary Figs. 2e, f). These findings indicate that a heat-labile proteinaceous factor of 30–50 kDa secreted by Δ*Hbl B. cereus* is a potent activator of the inflammasome.

### NHE activates the NLRP3 inflammasome

We previously excluded a contribution from NHE in driving the early inflammasome response owing to a role for HBL^31^. However, toxins have different kinetics and assembly processes, which determine their activity^39^. Therefore, we reasoned that NHE could have been overlooked as a potential candidate^34^. This idea is supported by our finding that another 30–50 kDa secreted factor is acting in a non-redundant manner in the activation of the NLRP3 inflammasome during *B. cereus* infection (Supplementary Fig. 2e, f), and that NHE is comprised of three subunits, called A, B, and C, all of which are of 30–50 kDa in size^40–43^.

**Fig. 3:**
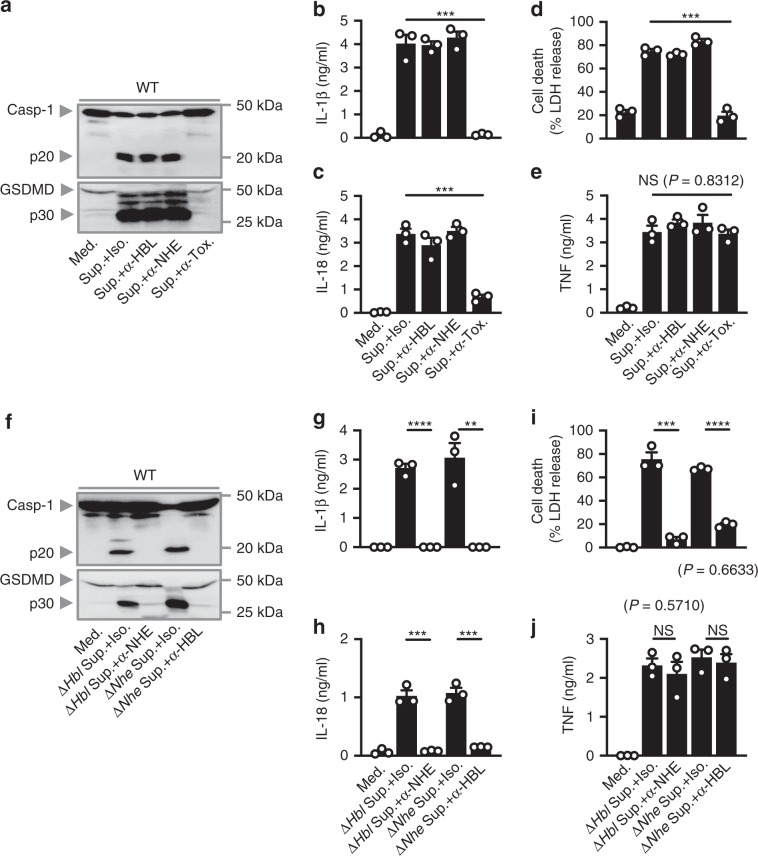
Neutralization of both HBL and NHE abrogates inflammasome activation. **a** Immunoblot analysis of caspase-1 and gasdermin D, of WT BMDMs left untreated or LPS-primed and assessed 20 h after treatment with the supernatant of WT *B. cereus* (Sup.) treated with an isotype control (Iso.), anti-HBL-neutralizing antibodies (α-HBL), anti-NHE-neutralizing antibodies (α-NHE) or with both anti-HBL- and anti-NHE-neutralizing antibodies (α-Tox.). Release of IL-1β **b**, and IL-18 **c** of BMDMs, death of BMDMs **d**, and release of TNF **e**, of BMDMs as treated in **a**. **f** Immunoblot analysis of caspase-1 and gasdermin D of WT BMDMs left untreated or LPS-primed and assessed 20 h after treatment with the supernatant of Δ*Hbl* treated with an isotype control or anti-NHE-neutralizing antibodies or assessed 20 h after treatment with the supernatant of Δ*Nhe B. cereus* (Δ*Nhe* Sup.) treated with an isotype control or anti-HBL-neutralizing antibodies. Release of IL-1β **g**, and IL-18 **h** of BMDMs, death of BMDMs **i**, and release of TNF **j** of BMDMs as treated in **f**. NS, not significant, **P < 0.01, ***P < 0.001, and ****P < 0.0001 (one-way ANOVA with Dunnett’s multiple-comparisons test **b**–**e** or student’s unpaired *t* test **g**–**j**. Each symbol represents an independent experiment **b**–**e** and **g**–**j**. Data are representative of three independent experiments (*n* = 3 in **a**–**j** mean and s.e.m. in **b**–**e**, and **g**–**j**). Source data are provided as a Source Data file.

To this end, we neutralized the supernatant of WT *B. cereus* with antibodies against NHE and/or HBL and stimulated WT BMDMs with the supernatant overnight. We found that neutralization of both NHE and HBL, but not NHE or HBL alone, abolished the ability of the supernatant to induce activation of caspase-1, cleavage of GSDMD, secretion of IL-1β and IL-18, and pyroptosis in WT BMDMs (Fig. 3a–e). Further, neutralization of NHE abrogated the ability of the supernatant of Δ*Hbl B. cereus* to induce activation of caspase-1, cleavage of GSDMD, secretion of IL-1β and IL-18, and pyroptosis in WT BMDMs (Fig. 3f–j). Conversely, neutralization of HBL abolished the ability of Δ*Nhe B. cereus* to elicit inflammasome responses in WT BMDMs (Fig. 3f–j). Neutralization of the supernatant with antibodies against either or both NHE and HBL did not affect the secretion of TNF and KC in BMDMs (Fig. 3e, j and Supplementary Fig. 2g, h), suggesting that secretion of inflammasome-independent cytokines was not compromised in response to supernatant treated with neutralizing antibodies. These data revealed that, in addition to blockade of HBL, abrogation of NHE by means of neutralization or genetic deletion prevented cytosolic inflammasome sensing of *B. cereus* infection.

**Fig. 4:**
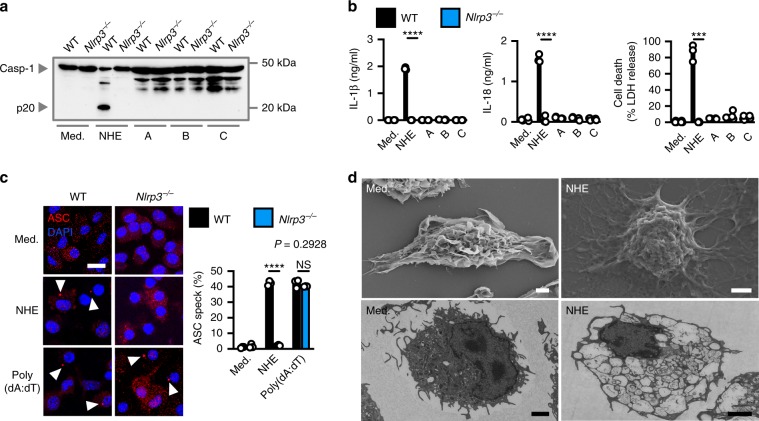
NHE activates the NLRP3 inflammasome. **a** Immunoblot analysis of caspase-1 of WT or *Nlrp3*^−/−^ BMDMs left untreated (Med.) or LPS-primed and assessed 3 h after treatment with recombinant NHE, or with the individual NHE subunits; **b** release of IL-1β and IL-18, and death of WT or *Nlrp3*^−/−^ BMDMs as treated in **a**. **c**, Immunofluorescent ASC speck analysis (red) in WT or *Nlrp3*^−/−^ BMDMs left untreated or LPS-primed and assessed 3 h after stimulation with NHE or 5 h after transfection with poly(dA:dT). Quantification of ASC specks as a percentage of total cells (DAPI). At least 200 cells from each genotype were assessed. Arrowheads indicate ASC specks. **d** Scanning electron microscopy analysis (top) or transmission electron microscopy analysis (bottom) of WT BMDMs left untreated or LPS-primed and assessed 3 h after treatment with NHE. Scale bar, 12 μm **c**, 2 μm **d**. NS, not significant, ***P < 0.001 and ****P < 0.0001 (student’s unpaired *t* test **b** and **c**). Each symbol represents an independent experiment **b** and **c**. Data are representative of three independent experiments (*n* = 3 in **a**–**d**; mean and s.e.m. in **b** and **c**. Source data are provided as a Source Data file.

To investigate further, we expressed the three recombinant subunits, which comprise NHE: subunit A (41 kDa), subunit B (40 kDa), and subunit C (37 kDa)^43^. Treatment with recombinant NHE, but not individual subunits, resulted in robust activation of inflammasome responses in LPS-primed WT BMDMs but not in LPS-primed *Nlrp3*^*–*/–^ BMDMs (Fig. 4a, b). Importantly, treatment of LPS-primed WT and *Casp11*^*–*/–^ BMDMs with NHE resulted in inflammasome activation (Supplementary Fig. 3a, b), indicating that NHE-induced inflammasome responses were not owing to NHE-mediated translocation of extracellular LPS into the cytoplasm to activate the caspase-11-NLRP3 pathway. Further, NHE-induced ASC speck formation in WT BMDMs but not in *Nlrp3*^*–*/–^ BMDMs, whereas dsDNA poly(dA:dT) induced ASC speck formation in both WT and *Nlrp3*^*–*/–^ BMDMs (Fig. 4c). In addition, we observed that in response to NHE, BMDMs lacking the full pore-forming ability of the pyroptotic factor GSDMD (*Gsdmd*^I105N/I105N^)^10,44,45^ had an impaired ability to secrete IL-1β and IL-18 and undergo cell death at 1 and 2 h, compared with WT BMDMs (Supplementary Fig. 3c, d). Electron microscopy analyses revealed that stimulation with NHE led to a disruption of cellular morphology and architecture in BMDMs (Fig. 4d and Supplementary Fig. 3e). Further, we found that NHE-induced secretion of IL-1β and IL-18 and led to cell death in human THP-1 monocytes, and that this response was impaired following inhibition of NLRP3 by MCC950 (Supplementary Fig. 3f). These findings highlight that NHE is a non-redundant toxin driving activation of the NLRP3 inflammasome during *B. cereus* infection, and that innate immune recognition of NHE by the NLRP3 inflammasome is functionally conserved between mice and humans.

### NHE assembles in a linear order on the plasma membrane

**Fig. 5:**
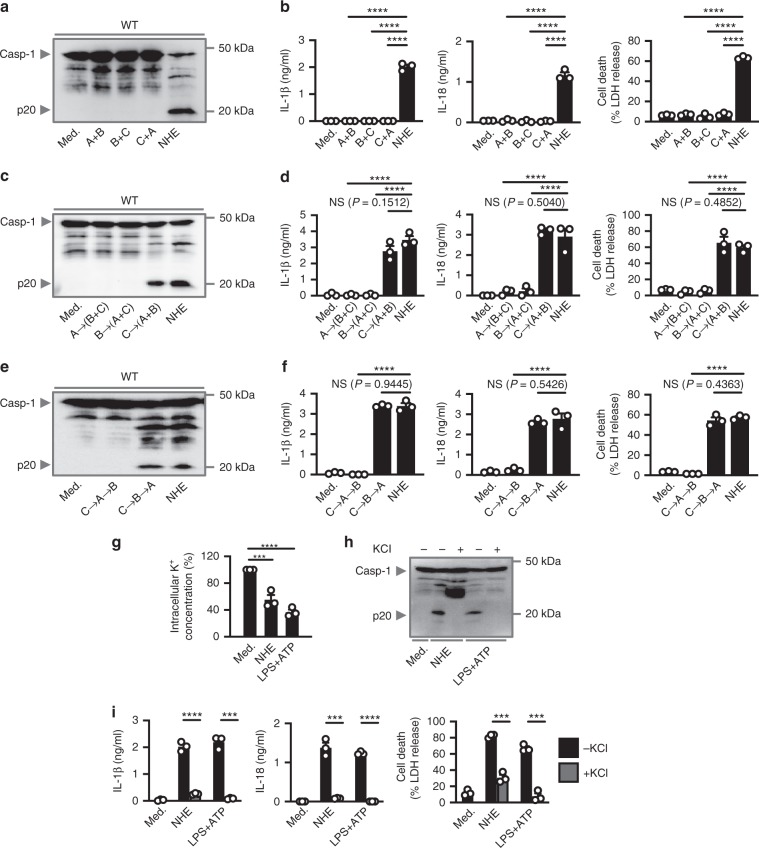
Linear assembly of NHE induces K^+^ efflux and activation of the NLRP3 inflammasome. **a** Immunoblot analysis of caspase-1 of WT BMDMs left untreated or LPS-primed and assessed 3 h after treatment with binary combinations of NHE, or after treatment with tripartite NHE. **b** Release of IL-1β and IL-18, and death of WT BMDMs as treated in **a**. **c**, **e** Immunoblot analysis of caspase-1 of WT BMDMs left untreated or LPS-primed and assessed 3 h after treatment with NHE component added in various orders. **d**, **f** Release of IL-1β and IL-18, and death of WT BMDMs as treated in **c** and **e**. **g** Inductively coupled plasma-optical emission spectrometry analysis of intracellular concentrations of K^+^ of BMDMs left untreated or LPS-primed and assessed 2 h after treatment with NHE, or 30 mins after treatment with ATP. **h** Immunoblot analysis of caspase-1 of WT BMDMs left untreated or LPS-primed and assessed 3 h after treatment with NHE, or 30 mins after treatment with ATP, in the absence (−) or presence (+ ; 50 mM) of extracellular KCl. **i** Release of IL-1β and IL-18, and death of WT BMDMs as treated in **h**. NS, not significant, ***P < 0.001 and ****P < 0.0001 (one-way ANOVA with Dunnett’s multiple-comparisons test **b**, **d**, **f**, and **g** or student’s unpaired *t* test **i**). Each symbol represents an independent experiment **b**, **d**, **f**, **g**, and **i**. Data are representative of three independent experiments (*n* = 3 in **a**–**i**; mean and s.e.m. in **b**, **d**, **f**, **g**, and **i**). Source data are provided as a Source Data file.

The tripartite composition of NHE suggests that an important assembly process must occur in order to exert the full activity of this toxin. Indeed, we found that individual components of NHE or all possible combinations of two of the three components of NHE did not trigger activation of the NLRP3 inflammasome (Figs. 4a, b and 5a, b), suggesting that all three subunits of NHE are essential in eliciting activation of the NLRP3 inflammasome. An earlier study reported that the B subunit is the binding component of the NHE complex on Vero cells^41^. A later study found that both the B and C subunits can bind to Vero cells, whereas the A subunit exerts cytotoxic effects in these cells^42^. A further study revealed that the C subunit initiates binding on Chinese Hamster Ovary cells^43^.

To investigate whether a specified order of assembly of NHE components leads to activation of the NLRP3 inflammasome in BMDMs, we stimulated WT BMDMs either with A, B, or C for 30 min, followed by extensive washing to remove unbound subunits prior to addition of the remaining subunits. We observed that C→A + B (where the arrow symbol indicates a washing step and the plus symbol indicates subunits added in concert) induced activation of caspase-1, secretion of IL-1β and IL-18, and pyroptosis (Fig. 5c, d). The order of A→B + C or B→A + C did not trigger inflammasome responses (Fig. 5c, d). These data suggest that C is the apical binding subunit. To elucidate the precise order of subunit assembly, leading to inflammasome activation, we treated the WT BMDMs with individual components in the order of C→A→B or C→B→A. We identified that the order of C→B→A led to activation of caspase-1, secretion of IL-1β and IL-18, and pyroptosis (Fig. 5e, f). These findings demonstrate that individual subunits of NHE follow a specific and linear order of NHE complex assembly to induce effective engagement of the NLRP3 inflammasome.

### NHE induces activation of NLRP3 via the efflux of K^+^

To assess whether NHE is maintained on the plasma membrane, leading to activation of the NLRP3 inflammasome, we treated WT BMDMs with NHE and performed immunofluorescence staining using antibodies against individual subunits of NHE. Immunofluorescence staining techniques revealed that all three subunits of NHE colocalized with the cell surface marker CD11b of BMDMs, suggesting that NHE localizes on the plasma membrane (Supplementary Fig. 4a–c). Further, we separated the cell membrane and cytosolic fractions of BMDMs stimulated with the supernatant of Δ*Hbl B. cereus* and found all three subunits of NHE in the membrane fraction (Supplementary Fig. 4d). In addition, inhibition of phagocytosis using the inhibitor cytochalasin B or cytochalasin D did not impede the ability of NHE, the supernatant of Δ*Hbl B. cereus*, or live Δ*Hbl B. cereus* to induce activation of the inflammasome in WT BMDMs (Supplementary Fig. 5a–d). These results indicate that internalization of NHE into BMDMs is not required to drive inflammasome activation. Indeed, transfection of NHE subunits into WT BMDMs did not trigger inflammasome activation, whereas transfection of flagellin triggered activation of the inflammasome (Supplementary Fig. 5e, f). These data suggest that localization of NHE on the plasma membrane is sufficient to induce activation of the inflammasome.

The localization of NHE implies that the mechanism driving activation of the NLRP3 inflammasome might emanate from the plasma membrane. NLRP3 is activated by membrane-induced cellular perturbations via pathways dependent on K^+^ efflux or independent of K^+^ efflux^46–50^. To investigate the role of K^+^ efflux in NHE-induced activation of the inflammasome, we used inductively coupled plasma-optical emission spectroscopy (ICP-OES) to measure the cytosolic concentration of K^+^ in WT BMDMs stimulated with NHE. The level of cytosolic concentration of K^+^ in BMDMs treated with NHE decreased substantially compared with untreated BMDMs, a reduction which is similar to that seen in BMDMs treated with the classical NLRP3 activator LPS + ATP (Fig. 5g). To confirm this observation, we added KCl into the cell culture media prior to addition of NHE. We observed that addition of extracellular K^+^ inhibited activation of the inflammasome in response to NHE, the supernatant of Δ*Hbl B. cereus*, Δ*Hbl B. cereus*, or LPS + ATP (Fig. 5h, i and Supplementary Fig. 6a, b). Further, we observed no difference in the cytosolic concentration of Na^+^ in BMDMs treated with NHE or LPS + ATP compared with untreated BMDMs (Supplementary Fig. 6c), and that addition of extracellular Na^+^ did not inhibit activation of the inflammasome in response to NHE or LPS + ATP (Supplementary Fig. 6d, e). These data suggest that localization of NHE on the plasma membrane is sufficient to induce activation of the inflammasome via efflux of K^+^.

### NHE forms pores in the lipid bilayer and is cytotoxic

**Fig. 6:**
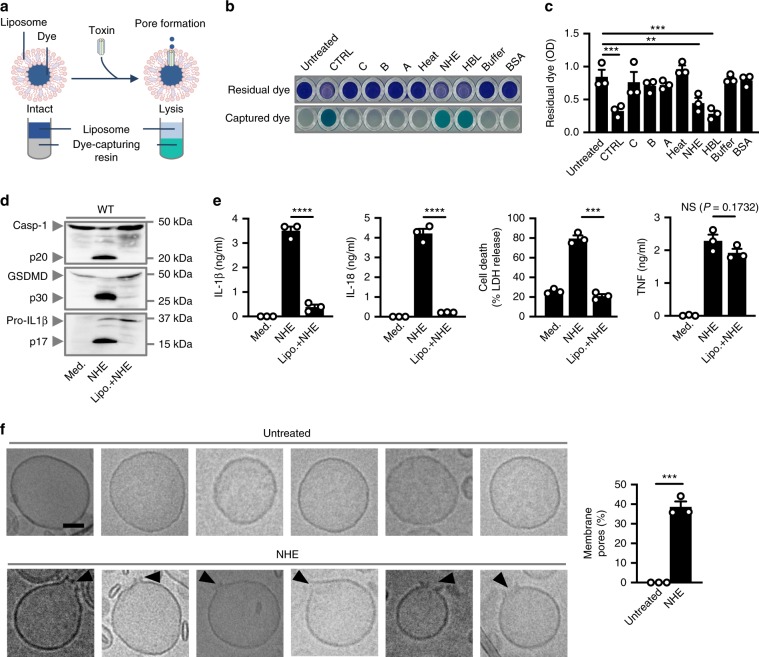
NHE forms pores in the cell membrane. **a** Schematic of toxin-induced liposomal rupture and leakage of dye. **b** Colorimetric analysis of liposomes left untreated, sonicated for 5 mins at 100 amplitude (CTRL) or assessed 30 mins after treatment with individual NHE subunits (C, B, or A), heat-inactivated NHE (Heat), NHE, HBL, buffer, or bovine serum albumin (BSA). **c** The absorbance (OD) of residual dye following treatment as in **b**. **d** Immunoblot analysis of caspase-1, gasdermin D, and IL-1β of WT BMDMs left untreated or LPS-primed and assessed 3 h after treatment with NHE in the absence or presence of liposomes (Lipo.). **e** Release of IL-1β, IL-18, and TNF, and death of WT BMDMs as treated in **d**. **f** Cryo-transmission electron microscopy analysis of liposomes left untreated (*n* = 760) or assessed 1 h after treatment with NHE (*n* = 800). Quantification of membrane pores of liposomes. Arrowheads indicate pores **f**. Scale bar, 50 nm **f**. NS, not significant, **P < 0.01, ***P < 0.001, and ****P < 0.0001 (one-way ANOVA with Dunnett’s multiple-comparisons test **c** or student’s unpaired *t* test **e**, **f**). Each symbol represents an independent experiment **c**, **e**, **f**. Data are representative of three independent experiments (*n* = 3 in **b**–**f**; mean and s.e.m. in **c**, **e**, and **f**). Source data are provided as a Source Data file.

Given that NHE subunits localize on the mammalian plasma membrane and induce K^+^ efflux, we hypothesized that NHE might be able to create a membrane pore, destabilizing the plasma membrane of BMDMs. To test this possibility, we formulated liposomes composed of the lipid species DPPC, DSPC and DOPC, and cholesterol, which mimic the mammalian plasma membrane^51^. These liposomes contain an internal aqueous methylene blue dye, which when released can be captured by dye-capturing resins (Fig. 6a). Addition of NHE, but not individual components of NHE, heat-inactivated NHE, or bovine serum albumin resulted in the release of dye from the liposomes (Fig. 6b, c). The level of dye leakage induced by NHE was similar to that of HBL or sonication of the liposomes (Fig. 6b, c). Further, incubation of NHE with liposomes inhibited the ability of NHE to induce inflammasome responses in BMDMs (Fig. 6d, e), suggesting potential sequestration of NHE on the membrane of the liposome.

**Fig. 7:**
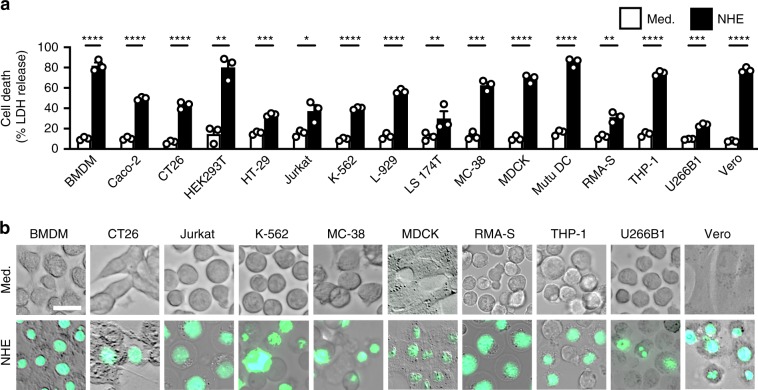
NHE induces cell death in multiple cell lineages from different species. **a** Death of various cell types left untreated or assessed 3 h after treatment with NHE. **b** Brightfield and fluorescent (SYTOX) images of cell lines left untreated or assessed 3 h after treatment with NHE. Green indicates dead cells **b**. Scale bar, 20 μm **b**. *P < 0.05, **P < 0.01, ***P < 0.001, and ****P < 0.0001 (student’s unpaired *t* test **a**). Each symbol represents an independent experiment **a**. Data are representative of three independent experiments (*n* = 3 in **a**, **b**; mean and s.e.m. in **a**). Source data are provided as a Source Data file.

Under cryo-transmission electron microscopy (Cryo-TEM), we observed distinct pores in the membrane of liposomes treated with NHE (Fig. 6f). The ability of NHE to insert into the membrane suggests that this toxin could kill different cell types from different host species. To this end, we treated immune and non-immune cells with NHE and found that NHE-induced cell death in all cell types tested, including those from humans, mice, monkeys, and dogs (Fig. 7a, b). To further explore the dynamics of NHE-induced cell death, we treated BMDMs with NHE and examined cell viability kinetics using IncuCyte assays. A lower concentration of 100 nM NHE-induced activation of caspase-1, cleavage of GSDMD and pro-IL-1β, secretion of IL-1β and IL-18, and cell death in WT BMDMs, but not in *Nlrp3*^*–*/–^ BMDMs (Supplementary Fig. 7a–c). However, a higher concentration of 500 nM NHE-induced rapid cell death in both WT and *Nlrp3*^*–*/–^ BMDMs within 20 min, but was unable to induce inflammasome responses (Supplementary Fig. 7a–c). Accumulation of NHE pores on the plasma membrane of BMDMs would mediate membrane rupture independently of the inflammasome, which provides an explanation for the mechanism of cell death in cell types which do not express inflammasome components (Fig. 7a, b). Taken together, these findings indicate that NHE induces pore formation in the cell membrane and can induce cytotoxic effects across multiple cell types and host species dictated by the bioavailability of the toxin.

### The putative transmembrane region of NHE-C is essential

**Fig. 8:**
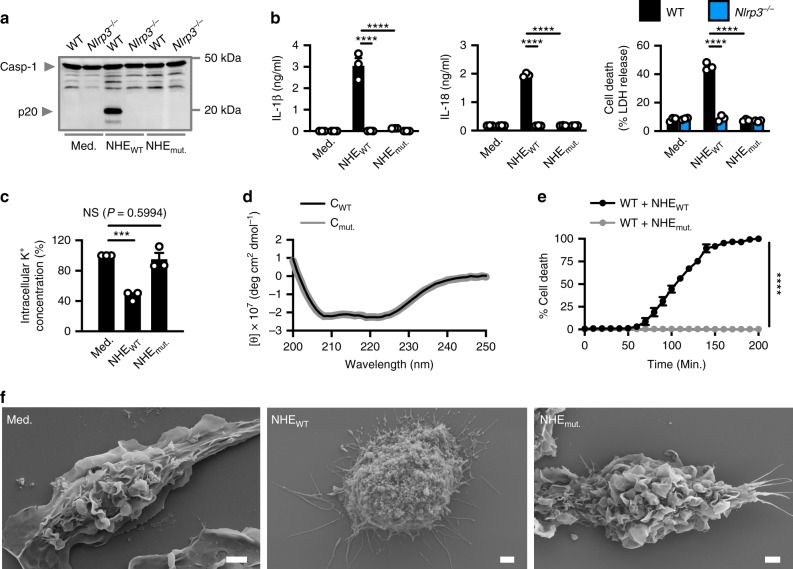
The transmembrane domain in NHE-C is essential for inflammasome activation and cell death. **a** Immunoblot analysis of caspase-1 of WT or *Nlrp3*^−/−^ BMDMs left untreated (Med.) or LPS-primed and assessed 3 h after treatment with C + B + A (NHE_WT_) or C_mut._ + B + A (NHE_mut._). **b** Release of IL-1β and IL-18, and death of WT or *Nlrp3*^−/−^ BMDMs as treated in **a**. **c** Inductively coupled plasma-optical emission spectrometry analysis of intracellular concentrations of K^+^ of BMDMs left untreated or assessed 3 h after treatment with NHE_WT_ or NHE_mut._. **d** Circular dichroism analysis of the secondary structures of C_WT_ or C_mut._. **e** IncuCyte live-imaging analysis of the viability of WT BMDMs left untreated or LPS-primed and assessed after treatment with NHE_WT_ or NHE_mut._. **f** Scanning electron microscopy analysis of WT BMDMs left untreated or LPS-primed and assessed 3 h after treatment with NHE_WT_ or NHE_mut._. Scale bar, 2 μm **f**. NS, not significant, ***P < 0.001 and ****P < 0.0001 (one-way ANOVA with Dunnett’s multiple-comparisons test **b**, **c** or Student’s unpaired *t* test **e**). Each symbol represents an independent experiment **b**, **c**, and **e**. Data are representative of three independent experiments (*n* = 3 in **a**–**c**, **e**, and **f**; *n* = 1 in **d**; mean and s.e.m. in **b**, **c**, and **e**. Source data are provided as a Source Data file.

Our data suggested that NHE is localized to the plasma membrane of macrophages and that the C subunit of NHE is the apical subunit binding the plasma membrane. Bioinformatic analysis of the protein sequence of the C subunit of NHE revealed the presence of a putative transmembrane region between amino-acid positions 228 and 250 (Supplementary Fig. 8a, b), consistent with previous studies^52,53^. We hypothesized that deletion of the putative transmembrane region in this subunit would abolish the ability of NHE to bind the plasma membrane and induce activation of the inflammasome. To address this hypothesis, we expressed the C subunit with and without the putative transmembrane region (Supplementary Fig. 8c). Next, we treated WT BMDMs with WT NHE (designated as NHE_WT_, carrying all the three WT subunits) or mutant NHE (designated as NHE_mut._, where the WT C subunit is replaced with the mutant C subunit). We found that NHE_WT_ induced K^+^ efflux, activation of caspase-1, secretion of IL-1β and IL-18, and pyroptosis, whereas NHE_mut._ did not (Fig. 8a–c). This observation was not owing to differences in secondary structures between the WT C subunit and mutant C subunit as shown by circular dichroism analysis (Fig. 8d). Further, IncuCyte, scanning electron microscopy, and transmission electron microscopy analyses collectively revealed that BMDMs treated with NHE_WT_, but not NHE_mut._, underwent cell death (Fig. 8e, f and Supplementary Fig. 9a, b).

**Fig. 9:**
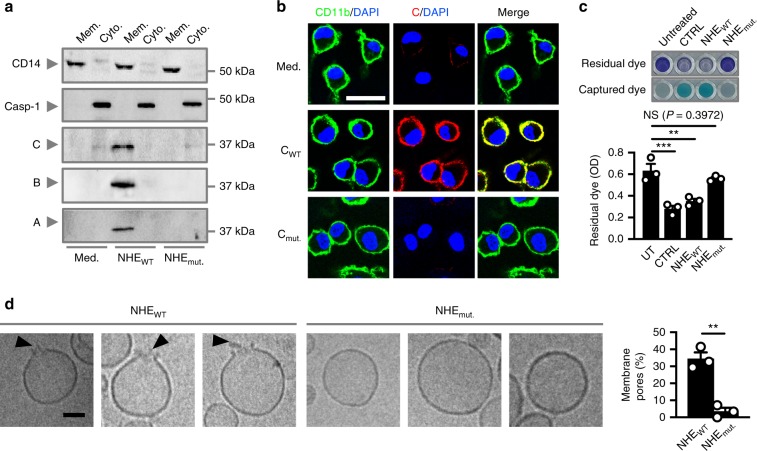
The transmembrane domain in NHE-C mediates membrane binding and pore formation. **a** Immunoblot analysis of CD14, caspase-1, C, B, and A of unprimed WT BMDMs left untreated or assessed 1 h after treatment with NHE_WT_ or NHE_mut._. Mem, membrane fraction; Cyto, cytosolic fraction. **b** Immunofluorescent analysis of CD11b (green) and C (red) in unprimed WT BMDMs left untreated or assessed 1 h after treatment with C_WT_ or C_mut._. **c** Colorimetric analysis (top) and the absorbance (OD, bottom) of liposomes left untreated, sonicated for 5 mins at 100 amplitude (CTRL) or assessed 30 mins after treatment with NHE_WT_ or NHE_mut._. **d** Cryo-transmission electron microscopy analysis of liposomes assessed 1 h after treatment with NHE_WT_ (*n* = 451) or NHE_mut._ (*n* = 545). Quantification of membrane pores of liposomes (right). Arrowheads indicate pores **d**. Scale bar, 25 μm **b**, 50 nm **d**. NS, not significant, **P < 0.01, ***P < 0.001 (one-way ANOVA with Dunnett’s multiple-comparisons test **c** or Student’s unpaired *t* test **d**). Each symbol represents an independent experiment **c**, **d**. Data are representative of three independent experiments (*n* = 3 in **a**–**d**; mean and s.e.m. in **c**, **d**). Source data are provided as a Source Data file.

To assess the membrane-binding ability of the mutant C subunit, WT BMDMs were treated either with NHE_WT_ or NHE_mut._ followed by fractionation of the membrane and cytosolic compartments. All three subunits of NHE were found in the membrane fraction of BMDMs treated with NHE_WT_, whereas none of the NHE subunits were detected in the membrane fraction of BMDMs treated with NHE_mut._ (Fig. 9a). Confocal microscopy techniques revealed that although the WT C subunit colocalized with CD11b on the plasma membrane, the mutant C subunit was unable to do so (Fig. 9b). Furthermore, we found that liposomes treated with NHE_WT_ underwent leakage of the dye, whereas liposomes treated with NHE_mut._ did not (Fig. 9c). Cryo-TEM analysis confirmed that liposomes treated with NHE_WT_ had membrane pores, whereas liposomes treated with NHE_mut._ were largely intact (Fig. 9d). Like NHE-C, the apical binding subunit of HBL, HBL-B, also carries a corresponding putative transmembrane region^31,54^. Importantly, deletion of the putative transmembrane region of HBL-B did not affect the secondary structure or the ability of this subunit to bind to the cell membrane of BMDMs (Supplementary Fig. 10a–d), suggesting a functional divergence of these putative transmembrane regions between the two toxins. These data collectively suggest that the putative transmembrane region of the C subunit of NHE might be important in mediating membrane binding and pore formation, leading to activation of the inflammasome.

### NHE induces inflammasome-mediated inflammation in the host

**Fig. 10:**
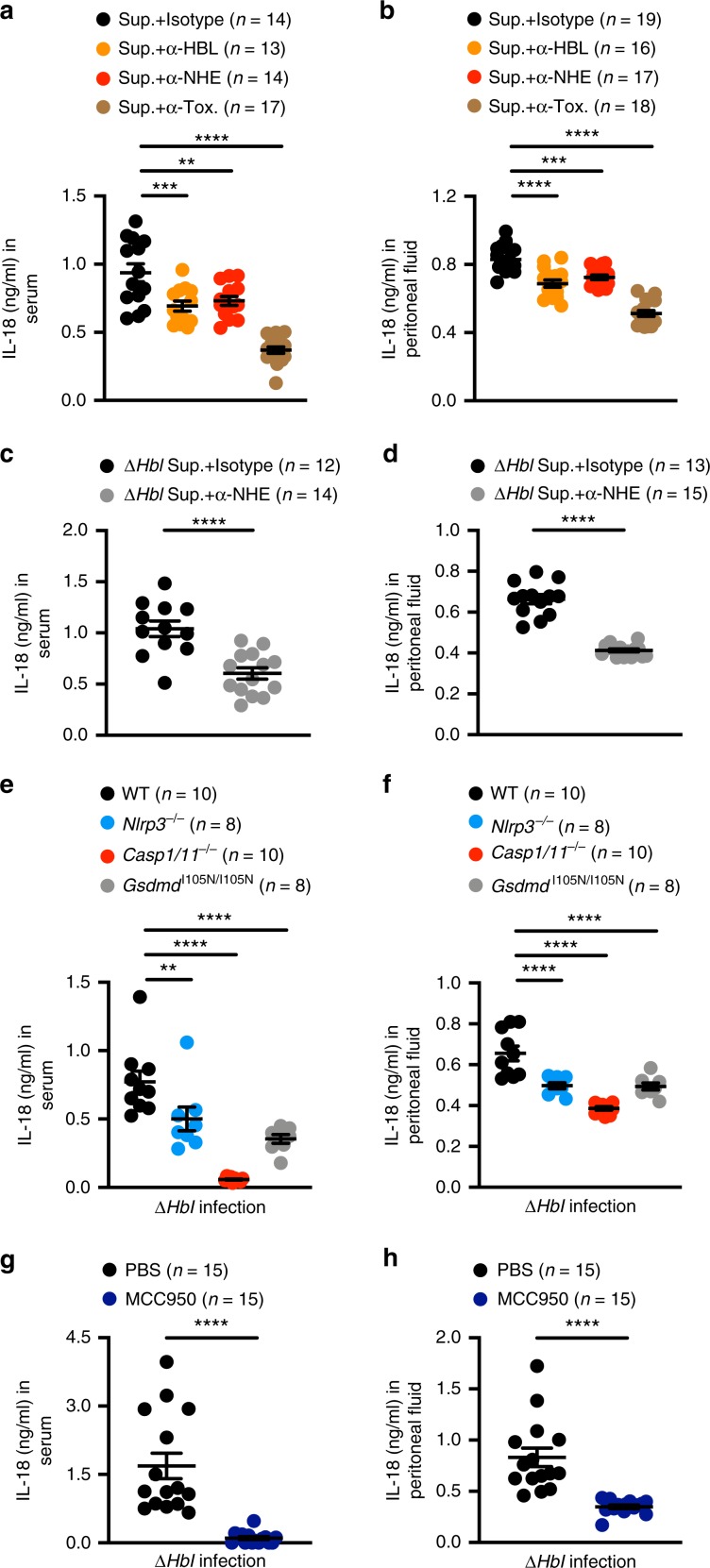
NHE induces activation of the NLRP3 inflammasome in the host. **a** Concentration of IL-18 in the serum of WT mice assessed 16 h after intraperitoneal injection with 200 μl of supernatant of WT *B. cereus* (Sup.), which had been treated with either an isotype control, anti-HBL-neutralizing antibodies (α-HBL), anti-NHE-neutralizing antibodies (α-NHE), or both (α-Tox.). **b** Concentration of IL-18 in the peritoneal fluid of WT mice assessed 1 h after intraperitoneal injection with treatment as in **a**. **c** Concentration of IL-18 in the serum of WT mice assessed 16 h after intraperitoneal injection with 200 μl Δ*Hbl B. cereus* supernatant, which had been treated with either an isotype control or anti-NHE-neutralizing antibodies (α-NHE). **d** Concentration of IL-18 in the peritoneal fluid of WT mice assessed 1 h after intraperitoneal injection with treatment as in **c**. **e** Concentration of IL-18 in the serum of WT, *Nlrp3*^−/−^, *Casp1/11*^−/−^ or *Gsdmd*^I105N/I105N^ mice assessed 6 h after intraperitoneal injection with 5 × 10^6^ CFU of Δ*Hbl B. cereus* (Δ*Hbl*). **f** Concentration of IL-18 in the peritoneal fluid of WT, *Nlrp3*^−/−^, *Casp1/11*^−/−^, or *Gsdmd*^I105N/I105N^ mice assessed 6 h after intraperitoneal injection with treatment as in **e**. **g** Concentration of IL-18 in the serum of WT mice administered with either PBS or MCC950, both intraperitoneally, followed by intraperitoneal infection with 5 × 10^6^ CFU of Δ*Hbl* with a corresponding second dose of PBS or MCC950, 6 h after infection. **h** Concentration of IL-18 in the peritoneal fluid of WT mice administered with either PBS or MCC950 (as in **g**) 6 h after infection with 5 × 10^6^ CFU of Δ*Hbl*. **P < 0.01, ***P < 0.001, and ****P < 0.0001 (one-way ANOVA with Dunnett’s multiple-comparisons test **a**, **b**, **e**, and **f** or Student’s unpaired *t* test **c**, **d**, **g**, and **h**. Each symbol represents an individual mouse **a**–**h**. Data are pooled from two independent experiments (*n* = 2 in **a**–**h**; mean and s.e.m. in **a**–**h**). Source data are provided as a Source Data file.

Given that NHE induces activation of the NLRP3 inflammasome in primary macrophages, we speculated that this toxin might induce activation of the NLRP3 inflammasome in the host. To test this idea, WT mice were administered with the supernatant of *B. cereus* or supernatant that had been neutralized against NHE, HBL, or both NHE and HBL. We observed that neutralization of both NHE and HBL abolished the ability of the supernatant to induce elevated amounts of IL-18 in the serum and peritoneal fluid of WT mice (Fig. 10a, b). Neutralization of either NHE or HBL alone led to some reduction in the secretion of IL-18 compared with isotype-treated controls; however, this reduction was less substantial compared to neutralization of both NHE and HBL (Fig. 10a, b). This finding suggests a potential synergistic effect of the two toxins in driving inflammasome responses. To validate that secretion of IL-18 was specific to NHE, WT mice were administered with the supernatant of Δ*Hbl B. cereus,* which had been neutralized with antibodies against NHE or with an IgG isotype. Indeed, neutralization of NHE impaired the ability of the supernatant of Δ*Hbl B. cereus* to induce secretion of IL-18 in WT mice compared with the corresponding control-treated supernatant (Fig. 10c, d).

To confirm a role for the NLRP3 inflammasome, we infected WT, *Nlrp3*^–/–^, *Casp1/11*^–/–^, and *Gsdmd*^I105N/I105N^ mice with Δ*Hbl B. cereus* and analyzed the levels of IL-18 in the circulation and peritoneal cavity. These analyses revealed that WT mice had elevated levels of IL-18, whereas *Nlrp3*^–/–^, *Casp1/11*^–/–^, and *Gsdmd*^I105N/I105N^ mice had reduced levels of IL-18 in response to Δ*Hbl B. cereus* infection (Fig. 10e, f). We speculate that the residual levels of IL-18 observed in *Nlrp3*^–/–^ and *Gsdmd*^I105N/I105N^ mice might be due to contributions from another inflammasome sensor or pyroptotic effector, respectively. Furthermore, pharmacological inhibition of the NLRP3 inflammasome with MCC950 attenuated secretion of IL-18 in the circulation and the peritoneal cavity of mice infected with Δ*Hbl B. cereus* (Fig. 10g, h). Together, these data indicate that recognition of NHE via the NLRP3 inflammasome provides another layer of innate immune mechanism in triggering inflammation in the host in response to *B. cereus* infection. The functional conservation between the mechanism of action of NHE and HBL is exploited by NLRP3 to ensure effective host recognition of the genetically diverse family of *B. cereus* strains, which express either one or both toxins.

## Discussion

*B. cereus* is a Gram-positive bacterium that causes gastrointestinal and extra-gastrointestinal pathologies in humans^1^. Our study reveals that cytosolic recognition of *B. cereus* infection by the host relies on exploitation of the conserved mechanism of action of two different toxins produced by the same bacterium (Supplementary Fig. 11a–c). Importantly, our results further suggest that NLRP3 might potentially exploit the functional and structural similarity of many other PAMPs. Studies have reported that NHE is found in 95–98% of the clinical, food, and environmental isolates of *B. cereus*^31,55,56^, and that HBL is found in 40–92% of the isolates^31,55,57,58^. The prevalence of NHE and HBL suggests that both toxins are key virulence factors important for the pathogenesis of *B. cereus* infection. Further, these epidemiological data give rise to scenarios that some strains might express NHE or HBL, or both NHE and HBL. We reasoned that the ability of macrophages to detect both toxins allows the host to respond to any naturally-occurring pathogenic variants and that a single NLR protein is able to sense any variants expressing at least one of the two toxins, or to sense strains that have phase-variable expression of the two toxins. An alternative perspective is that the expression of multiple functionally analogous toxins from the same bacterium is essential for infection in the host. Indeed, these toxins might be required for acquisition of nutrients and/or the destruction of immune cells during *B. cereus* infection. If this were not the case, it would have been evolutionarily advantageous to downregulate the expression of these toxins or removing these toxins altogether as a mechanism to avoid innate immune recognition.

The functional and structural similarity of NHE and HBL exploited by NLRP3 is based on pore formation and the permissiveness of the pore to K^+^ efflux. The ability of NHE to induce K^+^ efflux aligned with previous findings, suggesting that NHE induces the formation of large conducting openings that are permeable to cations^52,59^. Previous studies have classified NHE as a member of the α-helical pore-forming toxin family; members of which are characterized by the presence of an α-helical hydrophobic membrane-integrative region^52,53^. We observed that deletion of this hydrophobic region impairs the ability of subunit C to bind to membrane (Supplementary Fig. 11b), highlighting that the hydrophobic region in subunit C might be responsible for anchoring to the host membrane, similar to that observed in cytolysin A of the α-helical pore-forming toxin family^60,61^. These findings are consistent with a previous study, reporting that deletion of the same hydrophobic region in subunit C leads to a loss of cytotoxicity of NHE on Vero cells^42^. We postulate that the anchorage mediated by the hydrophobic region in subunit C can also function to initiate a series of conformational changes in subunit C, triggering recruitment of subunit B and subunit A for the final construction of the NHE pore complex. Although the NHE and HBL pores are both permissive to efflux of K^+^, subtle differences in the mechanism of action between the two toxins have been observed. Unlike subunit C of NHE, we found that deletion of the corresponding hydrophobic region of HBL-B, which is the apical subunit of HBL^31^, does not impair the ability of the subunit to bind the plasma membrane. It is possible that the hydrophobic region of HBL-B is not essential for membrane binding and that deletion of this region reduces steric hinderance to expose the bona fide-binding region/s of HBL-B, enhancing binding of HBL-B to the plasma membrane.

Targeting the conserved activity of bacterial pore-forming toxins might be efficacious against certain bacterial pathogens. For example, neutralization of the activity of the pore-forming toxin haemolysin A of *S. aureus*^62,63^ and the anthrax toxin of *B. anthracis*^64,65^, have been effective. These findings are of clinical importance given the increasing incidence of antibiotic-resistant bacteria, including *B. cereus*^1,66^. Therefore, a better understanding of the host defence strategy against *B. cereus* infection will be beneficial and neutralization of toxins might complement current therapies against infection caused by toxin-producing bacteria. In conclusion, we identified that the functional similarity and conserved mechanism of action of two multicomponent toxins of *B. cereus* is exploited for innate immune sensing by an inflammasome sensor protein. Further studies are required to understand whether NHE and HBL might target any cell surface receptor/s for binding to the plasma membrane.

## Methods

### Mice

C57BL/6 mice and *Gsdmd*^I105N/I105N^ mice^10^ were sourced from The Australian National University. *Nlrp3*^–/–^ ^67^, *Casp1/11*^–/–^ ^68^, and *Casp11*^–/–^ ^69^ mice were sourced from The Jackson Laboratory. *Nlrc4*^–/–^ ^70^ and *Asc*^–/–^ ^70^ mice were sourced from the University of Queensland. *Aim2*^–/–^ mice^71^ were sourced from Genentech. All mice are on, or backcrossed to, the C57BL/6 background for at least 10 generations. Male and female mice of 6–8 weeks old were used. Mice were bred and maintained at The Australian National University under specific pathogen-free conditions. All animal studies were conducted in accordance with the Protocol Number A2017/05 approved by The Australian National University Animal Experimentation Ethics Committee.

### BMDMs

Primary BMDMs were cultured for 6 days in Dulbecco's Modified Eagle Medium (DMEM) (11995073, ThermoFisher Scientific) supplemented with 10% fetal bovine serum (FBS; F8192, Sigma), 30% L929-conditioned media and 1% penicillin and streptomycin (10378016, Gibco ThermoFisher)^72^. BMDMs were seeded in antibiotic-free media at a concentration of 1 × 10^6^ cells per well in 12-well plates.

### Cell lines

CaCo-2 cells were cultured for 3 days in Eagle’s Minimum Essential Medium (EMEM) (670086, ThermoFisher Scientific) supplemented with 20% FBS, and 1% penicillin and streptomycin. CT26, Jurkat, RMA-S, and THP-1 cells were cultured for 3 days in Rosewell Park Memorial Institute (RPMI)-1640 (11875093, ThermoFisher Scientific) supplemented with 10% FBS, and 1% penicillin and streptomycin. HEK293T and HT-29 cells were cultured for 3 days in DMEM supplemented with 10% FBS, and 1% penicillin and streptomycin. L929, LS 174 T, MDCK, and Vero cells were cultured for 3 days in EMEM supplemented with 10% FBS, and 1% penicillin and streptomycin. K-562 cells were cultured for 3 days in Iscove's Modified Dulbecco's Medium (IMDM) (12440053, ThermoFisher Scientific) supplemented with 10% FBS, and 1% penicillin and streptomycin. MC-38 cells were cultured for 3 days in DMEM supplemented with 10% FBS, 1% non-essential amino acids (11140050, Gibco ThermoFisher), 1% 4-(2-hydroxyethyl)-1-piperazineethanesulfonic acid (HEPES; 15630080, Gibco ThermoFisher), and 1% penicillin and streptomycin. MutuDCs were cultured for 3 days in IMDM supplemented with 10% FBS, 1% sodium bicarbonate (25080094, Gibco ThermoFisher), 1% HEPES, and 1% penicillin and streptomycin. U266B1 cells were cultured for 3 days in RPMI supplemented with 15% FBS, and 1% penicillin and streptomycin. Cells were seeded in antibiotic-free media at a concentration of 0.5 × 10^6^ cells per well in 24-well plates.

### Bacterial culture

*B. cereus* was grown in Luria-Bertani (LB) media (244620, BD) overnight under aerobic conditions at 30 °C^31^. The *Bacillus* strains used in this study are listed in Supplementary Table 1. *Salmonella enterica* serovar Typhimurium was grown in LB media overnight under aerobic conditions at 37 °C. The next day, all bacteria were subcultured (1:10) in fresh media for 3-4 h.

### Stimulation of BMDMs with bacteria and inflammasome activators

Unprimed BMDMs were infected with WT or Δ*Hbl B. cereus* at multiplicity of infection (m.o.i.) of 2–5 for 3–20 h for inflammasome activation; BMDMs were infected with Δ*Hbl B. cereus* at m.o.i. of 2 for 0-60 min for pIkB, IkB, pERK, ERK, NLRP3, and GAPDH protein levels. To activate the canonical NLRP3 inflammasome, BMDMs were primed with 500 ng/ml ultrapure LPS from *E. coli* (ALX-581-014-L002, Enzo Life Sciences) for 4 h and stimulated with 5 mM ATP (10127531001, Roche) for 45 min. To activate the NLRC4 inflammasome, BMDMs were infected with *S*. *Typhimurium* at m.o.i. of 2 for 4 h. Ultrapure flagellin was mixed with 20 μl of the liposomal transfection reagent 1,2-Dioleoyl-3-trimethylammonium propane (DOTAP) (11202375001, Sigma) in phosphate-buffered saline (PBS). After 30 min, the complexes were added to LPS-primed BMDMs in Hank’s Balanced Salt Solution (HBSS, H9394, Sigma) and incubated for 3–5 h. To activate the AIM2 inflammasome, 2.5 μg of poly(dA:dT) (tlrl-patn, InvivoGen) were resuspended in PBS and mixed with 0.3 μl of Xfect polymer in Xfect reaction buffer (631318, Clontech Laboratories, Inc.). After 30 min, DNA complexes were added to BMDMs in Opti-MEM (31985-070, ThermoFisher Scientific) and left stimulated for 4 h. To activate caspase-11, 5 μg of LPS was transfected as described for poly(dA:dT) transfection. For inhibition studies, 50 μM of cytochalasin B (C6762, Sigma), 50 μM of cytochalasin D (C8273, Sigma), 20 μM of the selective and potent NLRP3 inhibitor, MCC950^36–38^, 50 mM of potassium chloride (KCl) (P9541, Sigma) or 50 mM of sodium chloride (NaCl) (S9888, Sigma) were added to BMDMs 30 min prior to stimulation. Cell culture supernatants were collected for enzyme-linked immunosorbent assay (ELISA) and lactate dehydrogenase assays. Cell culture supernatants and cell lysates were collected for immunoblot analysis.

### Stimulation of BMDMs with bacterial supernatant

The overnight bacterial culture was centrifuged at 4000 × *g* for 10 min. The supernatant was filter-sterilized using low-protein-binding 0.45-μm filters (SLHV033RS, Merck). For size-fractionation, the supernatant of Δ*Hbl B. cereus* was fractionated using spin-filter columns of the 9 kDa (89884 A, ThermoFisher Scientific), 30 kDa (UFC803096, Millipore), or 50 kDa range (UFC905096, Millipore). For heat inactivation, the supernatant of Δ*Hbl B. cereus* was heated to 50 °C, 75 °C or 100 °C for 10 min. To remove proteins, DNA or RNA, the supernatant of Δ*Hbl B. cereus* was pre-treated with Proteinase K (1 mg/ml, 19133, Qiagen), DNase I (1 mg/ml, Roche), or RNase A (1 mg/ml, Qiagen), respectively, for 1 h. In all, 50–150 μl of bacterial supernatant were added to LPS-primed BMDMs.

For neutralization studies, the supernatant from various *B. cereus* strains was treated with antibodies against NHE (2 μl of 0.8 mg/ml α-NHE-B), HBL (2 μl 0.8 mg/ml α-HBL-L_2_), or combination of NHE (2 μl of 0.8 mg/ml α-NHE-B) and HBL (2 μl 0.8 mg/ml α-HBL-L_2_) for 1 h before addition to LPS-primed BMDMs^43,55^. For controls, mouse isotype IgG1 (2 μl, 5415, Cell Signaling Technologies) was used.

### Cloning, protein expression and purification

Putative transmembrane regions in individual component of NHE were predicted using the software Membrane Protein Explorer server, also known as MPEx^73^. Helical wheel diagrams were constructed using the HELIQUEST server^74^. DNA sequences of the gene encoding NHE-C and HBL-B were acquired from the genome of *B. cereus* NVH 1230-88 (GenBank: CAB53340.2) and *B. cereus* strain ATCC 10876 (GenBank: CM000715.1) respectively, and were submitted to GenScript for cloning. In brief, DNA sequences encoding NHE-C and HBL-B with and without their putative transmembrane region were cloned into NcoI and XhoI restriction sites of the pET-28a(+)-TEV plasmid containing kanamycin resistance. The BL21(DE3) *E. coli* strain (C600003, ThermoFisher Scientific) was transformed with the pET-28a(+)-TEV plasmid vector. A single colony was picked to inoculate a starter culture of 10 mL LB_Kan_ broth (LB broth + 30 μg/ml kanamycin (10106801001, Roche)), which was incubated at 37 °C, shaking (180 rpm) overnight. After overnight incubation, 10 mL of starter culture was transferred into 1 L of LB_Kan_ broth and incubated at 37 °C, shaking (180 rpm) for 4 h until an OD_600_ of 0.6 was obtained. Expression was induced by adding isopropyl β-D-1-thiogalactopyranoside (1 mM; IPTG, Roche) and the incubation continued under identical conditions overnight. The culture suspension was centrifuged (5000 × *g*, 20 min, 4 °C) to pellet the bacteria and stored at −80 °C until required. The cell pellet was resuspended in lysis buffer (50 mM Tris, pH 8.0 (BP152-1, ThermoFisher Scientific); 300 mM NaCl (SA046, Chem-Supply); 5 mM imidazole (I2399, Sigma-Aldrich); 250 μg/ml lysozyme (89833, ThermoFisher Scientific); and 1 mM phenylmethylsulfonyl fluoride (78830, Sigma-Aldrich)). Cells were subsequently disrupted by sonication and centrifuged (27,000 × *g*, 20 min, 4 °C). The supernatant was passed through a 0.22 µm filter (SLGP033RS, Merck). The C-terminal hexa-His-tagged proteins were purified by using HisTrap HP histidine-tagged protein purification column (17524701, GE Healthcare Lifesciences) at 4 °C using the NGC Quest 10 Chromatography System (7880001, BioRad) and ChromLab software (BioRad). The target proteins were eluted from the HisTrap column using a stepwise gradient of imidazole (10% increments) from Buffer A (50 mM Tris, pH 8.0; 300 mM NaCl; 5 mM imidazole) to Buffer B (50 mM Tris, pH 8.0; 300 mM NaCl; 500 mM imidazole). Purity of the eluted proteins was analyzed by SDS-PAGE and Coomassie blue staining. The protein concentration was determined by spectrophotometric measurements of A280 using a NanoDrop 2000/2000c spectrophotometer.

### Circular dichroism measurement

The circular dichroism spectrum (*θ*) was recorded at 2 nm/s scanning speed on a Chirascan V100 Circular Dichroism Spectrometer from 200 to 250 nm at 25 °C. The mean residue ellipticity was calculated and the spectra are shown as an average of three scans.

### Stimulation of BMDMs with recombinant NHE and HBL

LPS-primed BMDMs were stimulated with all three NHE or HBL components, individually or in various combinations (NHE: 100 nM, HBL: 5 nM and 1–3 h for caspase-1 activation). For binding order studies, LPS-primed BMDMs were stimulated with a single NHE component (either A, or B, or C) for 1 h. This step was followed by extensive washing (denoted by: →) with PBS three times to remove unbound toxin. The two remaining components were added in concert (denoted by: +), or the second individual component was added for 1 h followed by the third individual component, with washing steps in between. For transfection of NHE, each reaction consisted of individual NHE components (100 nM each) or a combination of two components (50 nM each) mixed with 10 µl of the liposomal transfection reagent DOTAP. After 30 min the complexes were added to LPS-primed BMDMs in HBSS and left for 3 h.

### Liposome studies

Liposomes were synthesized^31^ and treated with NHE (30 nM), individual NHE component (30 nM), heat-inactivated NHE, HBL (0.5 µM), a protein buffer used to carry NHE and HBL (10 mM Tris HCl, 0.5 mM ethylenediaminetetraacetic acid (pH 8.0)), or bovine serum albumin (BSA) (1 μg/ml; 001000173, Jackson ImmunoResearch) or NHE_mut._ (30 nM). The liposomes were sonicated at 100 amplitude for 5 mins as control (CTRL). The released dye was captured by a cation exchanger resin Dowex (10–15 mg per well). The absorbance (OD) of residual dye was measured at a wavelength of 595 nm using the Infinite 200 PRO system (Tecan). To investigate the capacity of liposomes to inhibit NHE-induced activation of the inflammasome, recombinant NHE (100 nM) was left untreated or treated with liposomes (7 mM) for 1 h, prior to addition to LPS-primed BMDMs.

### Immunoblotting analysis

For caspase-1 immunoblotting, BMDMs and supernatant were lysed in lysis buffer and sample loading buffer containing sodium dodecyl sulphate (SDS) and 100 mM dithiothreitol (DTT). For immunoblotting of pIkB, IkB, pERK, ERK, NLRP3, and GAPDH, the supernatant was removed and BMDMs were washed once with PBS, followed by lysis in radioimmunoprecipitation buffer and sample loading buffer containing SDS and 100 mM DTT. Proteins were separated on 8–12% polyacrylamide gels. Following electrophoretic transfer of proteins onto polyvinyldifluoride (PVDF) membranes (IPVH00010, Millipore), membranes were blocked in 5% skim milk in tris-buffered saline with Tween-20 (TBST) and incubated overnight with primary antibodies against caspase-1 (1:1000 dilution, AG-20B-0042, Adipogen), pIkB (1:1000 dilution, 2859, Cell Signaling Technologies), IkB (1:1000 dilution, 9242, Cell Signaling Technologies), pERK (1:1000 dilution, 9101, Cell Signaling Technologies), ERK (1:1000 dilution, 9102, Cell Signaling Technologies), NLRP3 (1:1000 dilution, AG-20B-0014, Adipogen), GAPDH (1:10,000 dilution, 5174, Cell Signaling Technologies), CD14 (1:1000 dilution, 17000-1-AP, Proteintech), GSDMD (1:1000 dilution, ab209845, Abcam), IL-1β (1:1000 dilution, RDSAF401NA, R&D Systems), NHE-C (1:200 dilution, NHE-C rabbit antisera), NHE-B (1:1000 dilution, 1E11, 0.8 mg/ml), NHE-A (1:1000 dilution, 1G4, 0.8 mg/ml), or HBL-B (1:1000 dilution, HBL-B mouse antisera)^43,55^. PVDF membranes were then incubated with horseradish peroxidase-conjugated secondary antibody (1:5000) for 1 h and proteins were visualized using the Super Signal Femto substrate (34095, ThermoFisher Scientific) and the ChemiDoc Touch Imaging System (BioRad). Uncropped full length immunoblots are provided in the Source Data file.

### Immunofluorescence staining

Immunofluorescence stainings for visualization of inflammasomes were described in our previous publication^31^. For visualization of NHE and cell membrane, BMDMs were stimulated with NHE component C_WT_ or C_mut._ or C + B for 1 h, or C + B + A for 30 min, washed three times with PBS and fixed with 4% paraformaldehyde at room temperature for 15 min, followed by blocking in 1% BSA in PBS for 1 h. Cells were incubated with a rabbit sera anti-NHE-C (1:200 dilution in 1% BSA)^55^, a mouse anti-NHE-B, or -A (1:200 dilution in 1% BSA)^55^, an mouse anti-Histidine antibody (1:200 dilution in 1% BSA, ab18184, Abcam), and a rat fluorescein isothiocyanate-conjugated anti-CD11b antibody (1:200 dilution in 1% BSA, 101205, BioLegend) overnight at 4 **°**C. PBS containing 0.05% Tween-20 was used to wash between incubation steps. An anti-mouse secondary Rhodamine red antibody (115295146, Jackson ImmunoResearch) or rabbit secondary Rhodamine red antibody (111295144, Jackson ImmunoResearch) were used. The samples were mounted with VECTASHIELD Hardset Mounting Medium with DAPI (H-1500, Vector Laboratories, Inc.) and analyzed using a Leica SP5 confocal microscope.

### Scanning electron microscopy

BMDMs were washed with PBS and post-fixed with 2.5% glutaraldehyde in 0.1 M phosphate buffer overnight and further washed with PBS. Cells were fixed in 1% osmium tetroxide in double distilled water for 1 h and dehydrated in a series of alcohol. Dehydrated samples were dried using liquid carbon dioxide using critical point drying. Samples were then sputter-coated with platinum (3 nm thickness) at 15 mA for 2 min using the EMI TECH K550 Sputter coater and visualized under a Zeiss UltraPlus Field emission scanning electron microscope at 5 kV.

### Transmission electron microscopy

BMDMs were washed with PBS and post-fixed with 2.5% glutaraldehyde in 0.1 M phosphate buffer overnight and further washed with PBS. Cells were fixed in 1% osmium tetroxide in double distilled water for 1 h and dehydrated in a series of alcohol, and embedded in LR white resin (C023, ProSciTech). Samples were polymerized in a 60 °C oven overnight. Thin sections were cut at 80 nm and viewed using a Hitachi HA7100 transmission electron microscope at 100 kV.

### Cryo-transmission electron microscopy

Liposomes left untreated or treated with recombinant NHE_WT_ or NHE_mut._ (30 nM) for 1 h were applied on a holey carbon 400 mesh grid and left to adhere for 30 s. Grids were then blotted for 10 s to remove excess liposomes before plunge frozen using liquid ethane surrounded by a bath of liquid nitrogen. Samples were visualized under Hitachi 7100 TEM at 100 kv with a Cryo-TEM holder.

### Separation of membrane and cytosolic compartments

BMDMs were stimulated with the supernatant of Δ*Hbl B. cereus* (50 μl), recombinant NHE_WT_ or NHE_mut._ (100 nM), and HBL-B_WT_, or HBL-B_mut._ (5 nM) for 1 h, washed three times with PBS followed by separation of membrane and cytosolic fractions using the Mem-PER Plus Membrane Protein Extraction Kit according to the manufacturer’s instructions (89842, ThermoFisher Scientific).

### Lactate dehydrogenase assay

Levels of lactate dehydrogenase released by cells were determined using the CytoTox 96 Non-Radioactive Cytotoxicity Assay according to the manufacturer’s instructions (G1780, Promega).

### IncuCyte and cytotoxicity analysis

To track viability, BMDMs or various cell types were stimulated with 0.5 μM NHE (high) or 100 nM NHE (low), LPS + ATP, or with 100 nM NHE_WT_ or NHE_mut._, in presence of the SYTOX Green nuclear stain that penetrates compromised membranes (1 μM; S7020; Life Technologies). Cell death was monitored over 3 h using the IncuCyte Zoom imaging system (Essen Biosciences) or fluorescence was assessed using a Zeiss Axio Observer Fluorescent/Brightfield microscope.

### Real-time PCR analysis

RNA was extracted from BMDMs using TRIzol (15596018, ThermoFisher Scientific). The isolated RNA was converted into cDNA using the High-Capacity cDNA Reverse Transcription Kit (4368813, ThermoFisher). RT-PCR was performed on an ABI StepOnePlus System PCR instrument with SYBR Green Real Time PCR Master Mixes (4364346, ThermoFisher). Primer sequences can be found in Supplementary Table 2.

### Cytokine analysis

Cytokine concentrations from BMDMs were calculated using a multiplex ELISA (MCYTOMAG-70K, EMD Millipore) or IL-18 ELISA (BMS618-3TEN, ThermoFisher) according to the manufacturers’ instructions. Cytokine levels from THP-1 cells were calculated using a human IL-1β ELISA (BMS224-2TEN, ThermoFisher) or human IL-18 ELISA (BMS267-2TEN, ThermoFisher) according to the manufacturer’s instructions.

### ICP-OES analysis

The intracellular concentrations of K^+^ and Na^+^ ions were determined by ICP-OES analysis. In brief, BMDMs were stimulated with recombinant WT or mutant NHE for 2 h, washed three times with PBS followed by lysis with concentrated nitric acid (HNO_3_). The cell lysates were analysed for K^+^ and Na^+^ ions performed through ICP-OES using a PerkinElmer OPTIMA 7300 ICP Optical Emission Spectrometer (PerkinElmer).

### Animal infection

To investigate the role of NHE in inflammasome activation in vivo, WT mice were injected intraperitoneally with 200 μl of supernatant of WT or Δ*Hbl B. cereus,* which had been treated with either isotype control, anti-HBL-neutralizing antibodies, anti-NHE-neutralizing antibodies or both anti-HBL-neutralizing antibodies and anti-NHE-neutralizing antibodies. To investigate contributions from the NLRP3 inflammasome, mice were injected, via an i.p. route, with 5 × 10^6^ colony-forming units (CFUs) of culture of Δ*Hbl B. cereus*. To investigate the effects of MCC950^36–38^, mice were intraperitoneally injected with 50 mg per kg of MCC950 dissolved in PBS or with an equal amount of the vehicle control PBS. One hour later, mice were intraperitoneally injected with 5 × 10^6^ CFU of Δ*Hbl B. cereus* along with a second dose of 50 mg per kg of MCC950 or a second dose of PBS. For cytokine measurement serum and peritoneal fluid were collected after 1–16 h for analysis by ELISA.

### Statistical analysis

The GraphPad Prism 6.0 software was used for data analysis. Data are shown as mean ± s.e.m. Statistical significance was determined by *t* tests (two-tailed) for two groups or one-way analysis of variance (with Dunnett’s multiple-comparisons test) for three or more groups. *P* < 0.05 was considered statistically significant.

### Reporting summary

Further information on research design is available in the Nature Research Reporting Summary linked to this article.

## Supplementary information

Supplementary Information

Reporting Summary

## Data Availability

The data that support the findings of this study are included in this published article along with its Supplementary Information files, or are also available from the corresponding author upon request. The raw data used to make Fig. 1a–d; 2a, b, d; 3a–j; 4a–c; 5a–i; 6c–f; 7a; 8a–e; 9a, c, d; 10a–h and Supplementary Figs. 1b–f; 2a–h; 3a–d, f; 4d; 5a–f; 6a–e; 7a–c; and 10c, d are presented in the Source Data File.

## Source data

Source Data
