# Supplementary material for: Bacillus cereus non-haemolytic enterotoxin activates the NLRP3 inflammasome
Source: Nat Commun. 2020 Feb 6;11:760. doi: 10.1038/s41467-020-14534-3 (PMC7005308; doi:10.1038/s41467-020-14534-3)
Supplement: Supplementary file 1 — Supplementary Information [file 41467_2020_14534_MOESM1_ESM.pdf]

**_Bacillus cereus_ non-haemolytic enterotoxin activates the NLRP3 inflammasome**

Daniel Fox,[1†] Anukriti Mathur,[1†] Yansong Xue,[1] Yunqi Liu,[1] Wei Hong Tan,[1] Shouya Feng,[1] Abhimanu Pandey,[1] Chinh Ngo,[1] Jenni A. Hayward,[1] Ines I. Atmosukarto,[2] Jason D. Price,[2] Matthew D. Johnson,[3] Nadja Jessberger,[4] Avril A.B. Robertson,[5] Gaetan Burgio,[1] David C. Tscharke,[1] Edward M. Fox,[6] Denisse L. Leyton,[3,7] Nadeem O. Kaakoush,[8] Erwin Märtlbauer,[4] Stephen H. Leppla,[9] Si Ming Man[1*]

[1] *Department of Immunology and Infectious Disease, The John Curtin School of Medical Research, The Australian National University, Canberra, Australia.*

[2] *Lipotek Pty Ltd. The John Curtin School of Medical Research, The Australian National University, Canberra, Australia.*

[3] *Research School of Biology, The Australian National University, Canberra, Australia.*

[4] *Department of Veterinary Sciences, Faculty of Veterinary Medicine, Ludwig-Maximilians-Universität München, Oberschleißheim, Germany.*

[5] *School of Chemistry and Molecular Biosciences, The University of Queensland, Brisbane, Queensland 4072, Australia.*

[6] *Department of Applied Sciences, Northumbria University, Newcastle Upon Tyne, UK.*

[7] *Medical School, The Australian National University, Canberra, Australia.*

[8] *School of Medical Sciences, UNSW Sydney, Sydney, NSW, 2052, Australia.*

[9] *Microbial Pathogenesis Section, Laboratory of Parasitic Diseases, National Institute of Allergy and Infectious Diseases, National Institutes of Health, Bethesda, MD 20892, USA.*

[†] These authors contributed equally to this work

*Correspondence:

Si Ming Man

*Department of Immunology and Infectious Disease, The John Curtin School of Medical Research, The Australian National University, Canberra, 2601, Australia.*

Tel: (61) 2 612 56793

E-mail: siming.man@anu.edu.au

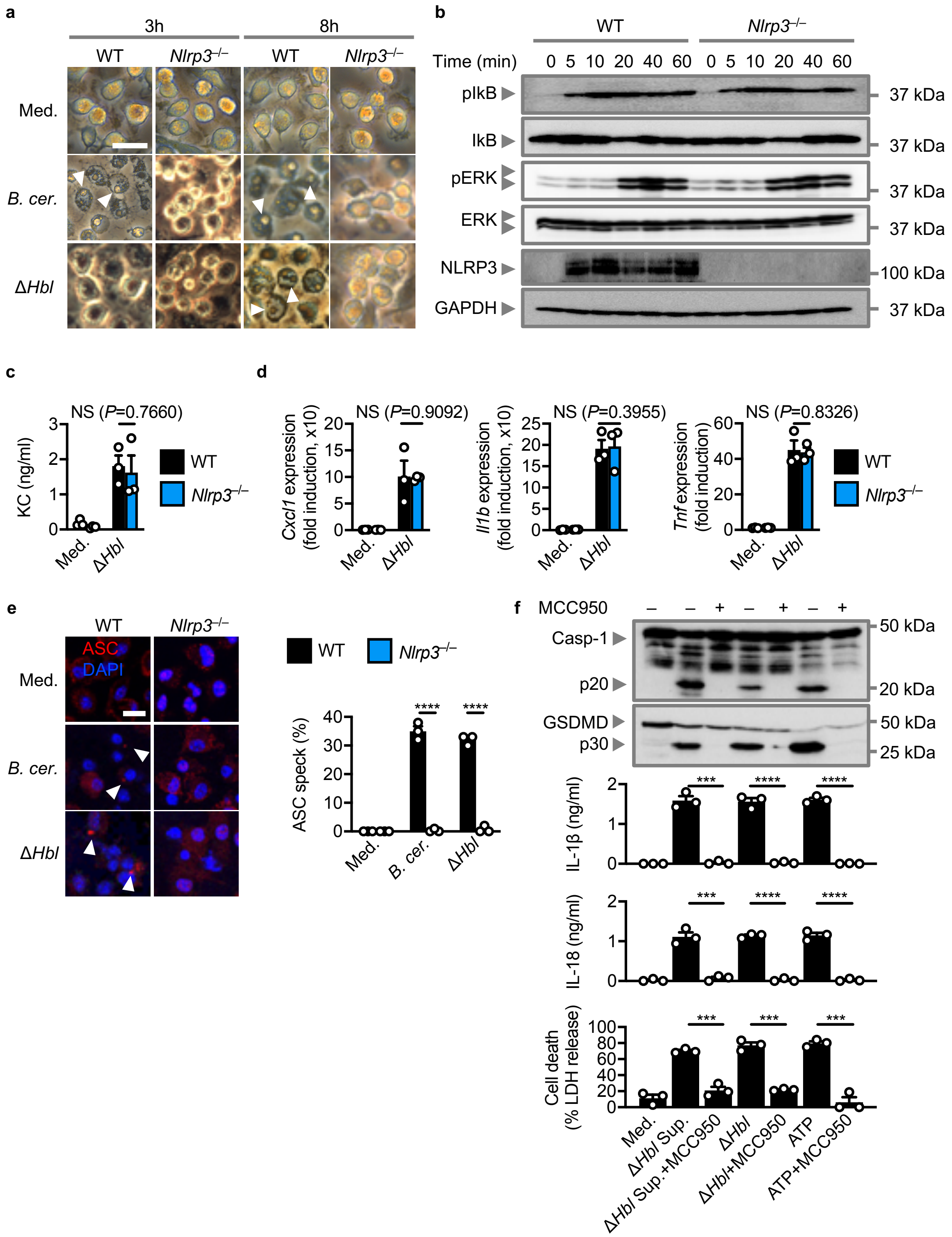
a
3h
8h
WT
Nlrp3–/–
WT
Nlrp3–/–
Med.
B. cer.
ΔHbl
b
WT
Nlrp3–/–
Time (min)
0 5 10 20 40 60 0 5 10 20 40 60
pIkB
IkB
pERK
ERK
NLRP3
GAPDH
37 kDa
37 kDa
37 kDa
37 kDa
100 kDa
37 kDa
c
NS (P=0.7660)
KC (ng/ml)
WT
Nlrp3–/–
Med.
ΔHbl
d
NS (P=0.9092)
Cxcl1 expression (fold induction, x10)
NS (P=0.3955)
Il1b expression (fold induction, x10)
NS (P=0.8326)
Tnf expression (fold induction)
WT
Nlrp3–/–
e
WT
Nlrp3–/–
ASC
DAPI
Med.
B. cer.
ΔHbl
WT
Nlrp3–/–
ASC speck (%)
****
****
f
MCC950
– – + – + – +
Casp-1
p20
GSDMD
p30
50 kDa
20 kDa
50 kDa
25 kDa
IL-1β (ng/ml)
IL-18 (ng/ml)
Cell death (% LDH release)
***
****
****
***
Med.
ΔHbl Sup.
ΔHbl Sup.+MCC950
ΔHbl
ΔHbl+MCC950
ATP
ATP+MCC950

**Supplementary Fig. 1 | Production of other pro-inflammatory cytokines is not affected by the absence of NLRP3.** **a**, Microscopy analysis of death of WT or *Nlrp3*$^{-/-}$ BMDMs left untreated (Med.) or assessed 3 h or 8 h after infection with WT (*B. cer.*, m.o.i. of 5) or Δ*Hbl B. cereus* (Δ*Hbl*, m.o.i. of 5) **b**, Immunoblot analysis of phospho-IkB (pIkB), IkB, phospho-ERK (pERK), ERK, NLRP3 and GAPDH of unprimed WT or *Nlrp3*$^{-/-}$ BMDMs 0-60 min after infection with Δ*Hbl B. cereus* (m.o.i. of 2). **c**, Release of KC in WT or *Nlrp3*$^{-/-}$ BMDMs left untreated or assessed 20 h after infection with Δ*Hbl B. cereus*. **d**, RT-PCR analysis of the gene encoding KC (*Cxcl1*), IL-1β and TNF in WT or *Nlrp3*$^{-/-}$ BMDMs 3 h after infection with Δ*Hbl B. cereus*, relative to *Gapdh*. **e**, Confocal microscopy analysis and quantification of ASC specks (red) in WT or *Nlrp3*$^{-/-}$ BMDMs left untreated or assessed 8 h after infection as in **a**. At least 200 BMDMs from each genotype were analyzed. **f**, Immunoblot analysis of caspase-1 and gasdermin D, release of IL-1β and IL-18, and death of BMDMs left untreated or LPS-primed and assessed 20 h after treatment with the supernatant of Δ*Hbl B. cereus* or 20 h after infection with Δ*Hbl B. cereus* (m.o.i. of 5) or 30 min after stimulation with ATP in absence or presence of MCC950 (20 μM). Scale bars, 25 μm (**a**), 12 μm (**e**). Arrowheads indicate dead cells (**a**) or inflammasome specks (**e**). Each symbol represents an independent experiment (**c**-**f**). NS, not statistically significant, $^{***}P < 0.001$ and $^{****}P < 0.0001$ [student's unpaired *t*-test (**c**, **d**, **e**, and **f**)]. Data are representative of three independent experiments ($n = 3$ in **a-f**; mean and s.e.m. in **c-f**). Source data are provided as a Source Data file.

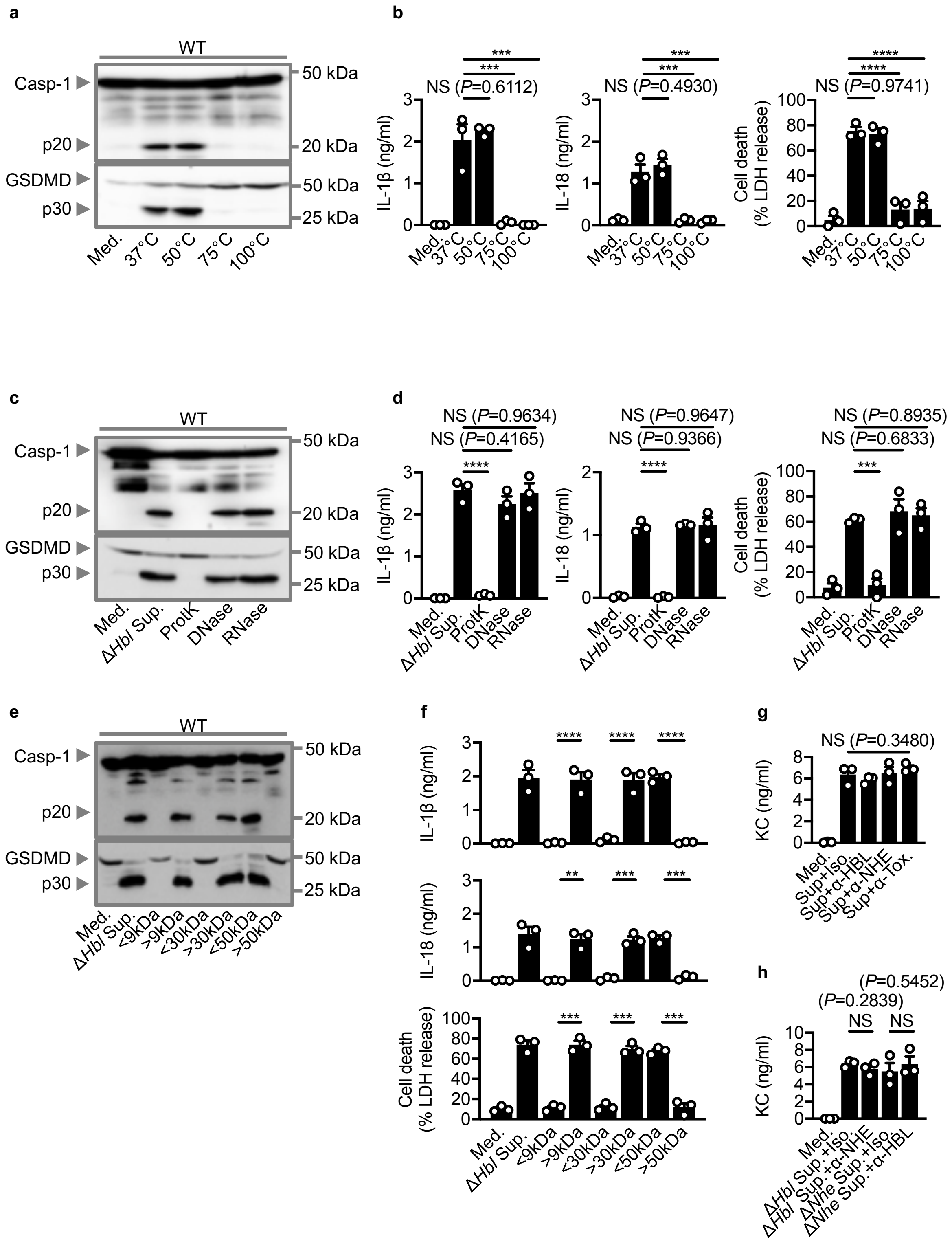
a
WT
Casp-1
p20
GSDMD
p30
50 kDa
20 kDa
50 kDa
25 kDa
Med.
37°C
50°C
75°C
100°C
b
NS (P=0.6112)
NS (P=0.4930)
NS (P=0.9741)
IL-1β (ng/ml)
IL-18 (ng/ml)
Cell death (% LDH release)
c
WT
Casp-1
p20
GSDMD
p30
Med.
ΔHbl Sup.
ProtK
DNase
RNase
d
NS (P=0.9634)
NS (P=0.4165)
NS (P=0.9647)
NS (P=0.9366)
NS (P=0.8935)
NS (P=0.6833)
e
WT
Med.
ΔHbl Sup.
<9kDa
>9kDa
<30kDa
>30kDa
<50kDa
>50kDa
f
g
NS (P=0.3480)
KC (ng/ml)
Med.
Sup+Iso
Sup+α-HBL
Sup+α-NHE
Sup+α-Tox.
h
(P=0.5452)
(P=0.2839)
NS
NS
ΔHbl Sup.+Iso
ΔHbl Sup.+α-NHE
ΔNhe Sup.+Iso
ΔNhe Sup.+α-HBL

**Supplementary Fig. 2 │ The secreted factor is a heat-labile protein of 30-50 kDa in size.** **a**, Immunoblot analysis of caspase-1 and gasdermin D of BMDMs left untreated (Med.) or LPS-primed and assessed 20 h after treatment with the supernatant of Δ*Hbl* (Δ*Hbl* Sup.) which had been treated with the indicated temperatures; **b**, Release of IL-1β and IL-18, and cell death of BMDMs treated as in **a**. **c**, Immunoblot analysis of caspase-1 and gasdermin D of BMDMs left untreated or LPS-primed and assessed 20 h after treatment with the supernatant of Δ*Hbl* which had been treated with proteinase K (ProtK), DNase or RNase; **d**, Release of IL-1β and IL-18, and cell death of BMDMs treated as in **c**. **e**, Immunoblot analysis of caspase-1 and gasdermin D of BMDMs left untreated or LPS-primed and assessed 20 h after treatment with the size-fractionated supernatant of Δ*Hbl*; **f**, Release of IL-1β and IL-18, and cell death of BMDMs treated as in **e**. **g**, Release of KC in WT BMDMs left untreated or LPS-primed and assessed 20 h after treatment with the supernatant of WT *B. cereus* (Sup.) treated with an isotype control (Iso.), or with neutralizing antibodies against HBL (α-HBL), NHE (α-NHE), or both (α-Tox.). **h**, Release of KC in WT BMDMs left untreated or LPS-primed and assessed 20 h after treatment with the supernatant of Δ*Hbl* treated with an isotype control or α-NHE, or assessed 20 h after treatment with the supernatant of Δ*Nhe B. cereus* (Δ*Nhe* Sup.) treated with an isotype control or α-HBL. Each symbol represents an independent experiment (**b**, **d**, and **f**). NS, not statistically significant, $^{**}P < 0.01$, $^{***}P < 0.001$ and $^{****}P < 0.0001$ [one-way ANOVA with Dunnett's multiple-comparisons test (**b**, **d** and **g**) or student's unpaired *t*-test (**f**, **h**)]. Data are representative three independent experiments ($n$ = 3 in **a-h**; mean and s.e.m. in **b**, **d**, **f-h**). Source data are provided as a Source Data file.

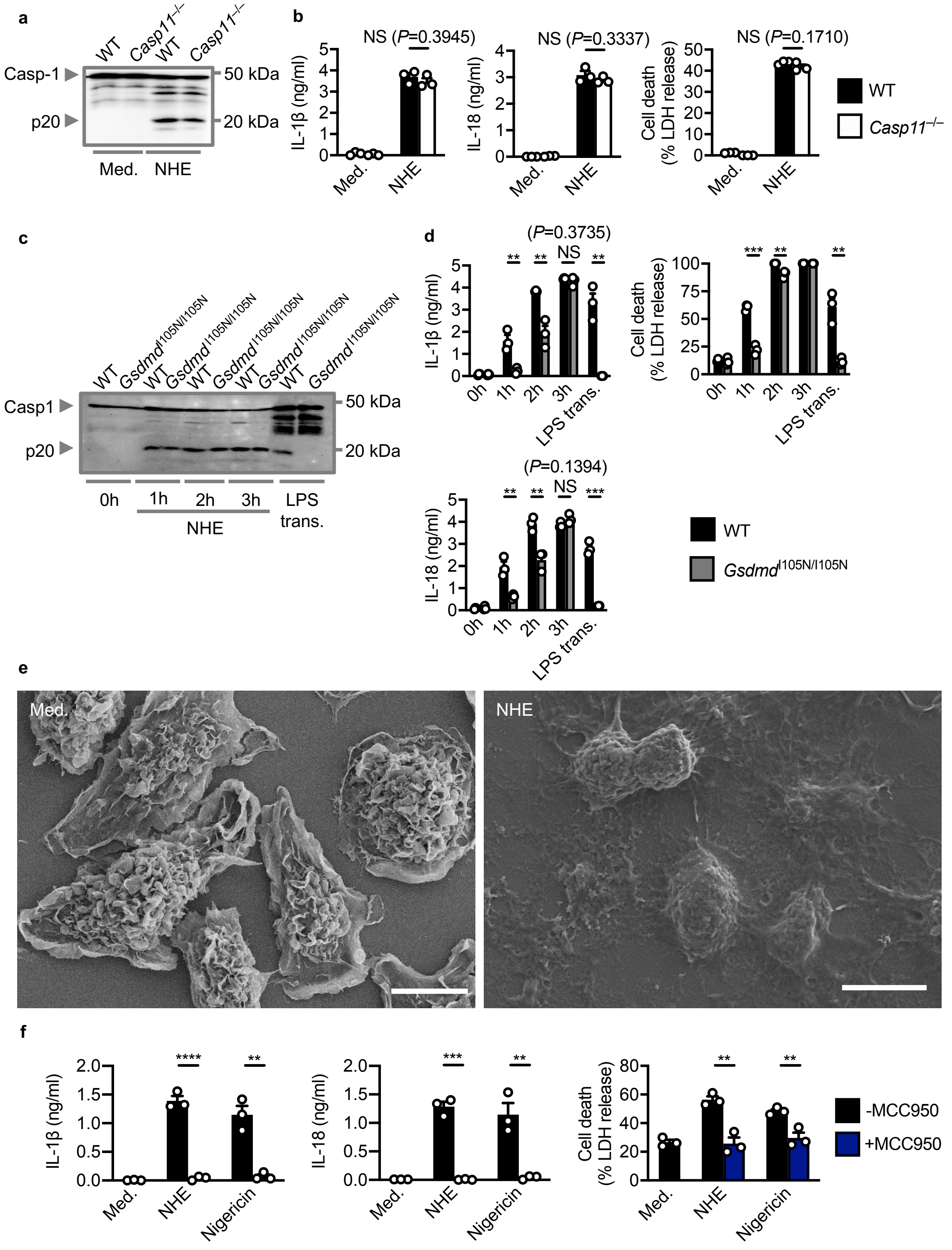
a
WT
Casp11−/−
WT
Casp11−/−
Casp-1
p20
50 kDa
20 kDa
Med.
NHE
b
NS (P=0.3945)
NS (P=0.3337)
NS (P=0.1710)
IL-1β (ng/ml)
IL-18 (ng/ml)
Cell death (% LDH release)
Med.
NHE
WT
Casp11−/−
c
WT
Gsdmd I105N/I105N
Casp1
p20
50 kDa
20 kDa
0h
1h
2h
3h
NHE
LPS trans.
d
(P=0.3735)
NS
(P=0.1394)
IL-1β (ng/ml)
Cell death (% LDH release)
IL-18 (ng/ml)
0h
1h
2h
3h
LPS trans.
WT
Gsdmd I105N/I105N
e
Med.
NHE
f
IL-1β (ng/ml)
IL-18 (ng/ml)
Cell death (% LDH release)
Med.
NHE
Nigericin
-MCC950
+MCC950

**Supplementary Fig. 3 │ NHE induces activation of the inflammasome in both mice and humans.** **a**, Immunoblot analysis of caspase-1 of WT and *Casp11*$^{-/-}$ BMDMs left untreated (Med.) or LPS-primed and assessed 3 h after treatment with NHE; **b,** Release of IL-1β and IL-18, and cell death of BMDMs as treated in **a**. **c,** Immunoblot analysis of caspase-1 of WT and *Gsdmd*$^{I105N/I105N}$ BMDMs left untreated (0 h) or LPS-primed and assessed at either 1, 2 or 3 h after treatment with NHE; **d**, Release of IL-1β and IL-18, and cell death of BMDMs as treated in **c**. **e**, Overview of scanning electron microscopy analysis of WT BMDMs left untreated or LPS-primed and assessed 2 h after stimulation with NHE. **f**, Release of IL-1β and IL-18, and death of WT THP-1 cells left untreated and assessed 3 h after treatment with NHE or Nigericin. Scale bars, 10 μm (**e**). Each symbol represents an independent experiment (**b**, **d**, and **f**). NS, not statistically significant, $^{**}P < 0.01$ and $^{***}P < 0.001$, $^{****}P < 0.0001$ [student's unpaired *t*-test (**b**, **d**, and **f**)]. Data are representative of three independent experiments (*n* = 3 in **a-f**; mean and s.e.m. in **b**, **d** and **f**). Source data are provided as a Source Data file.

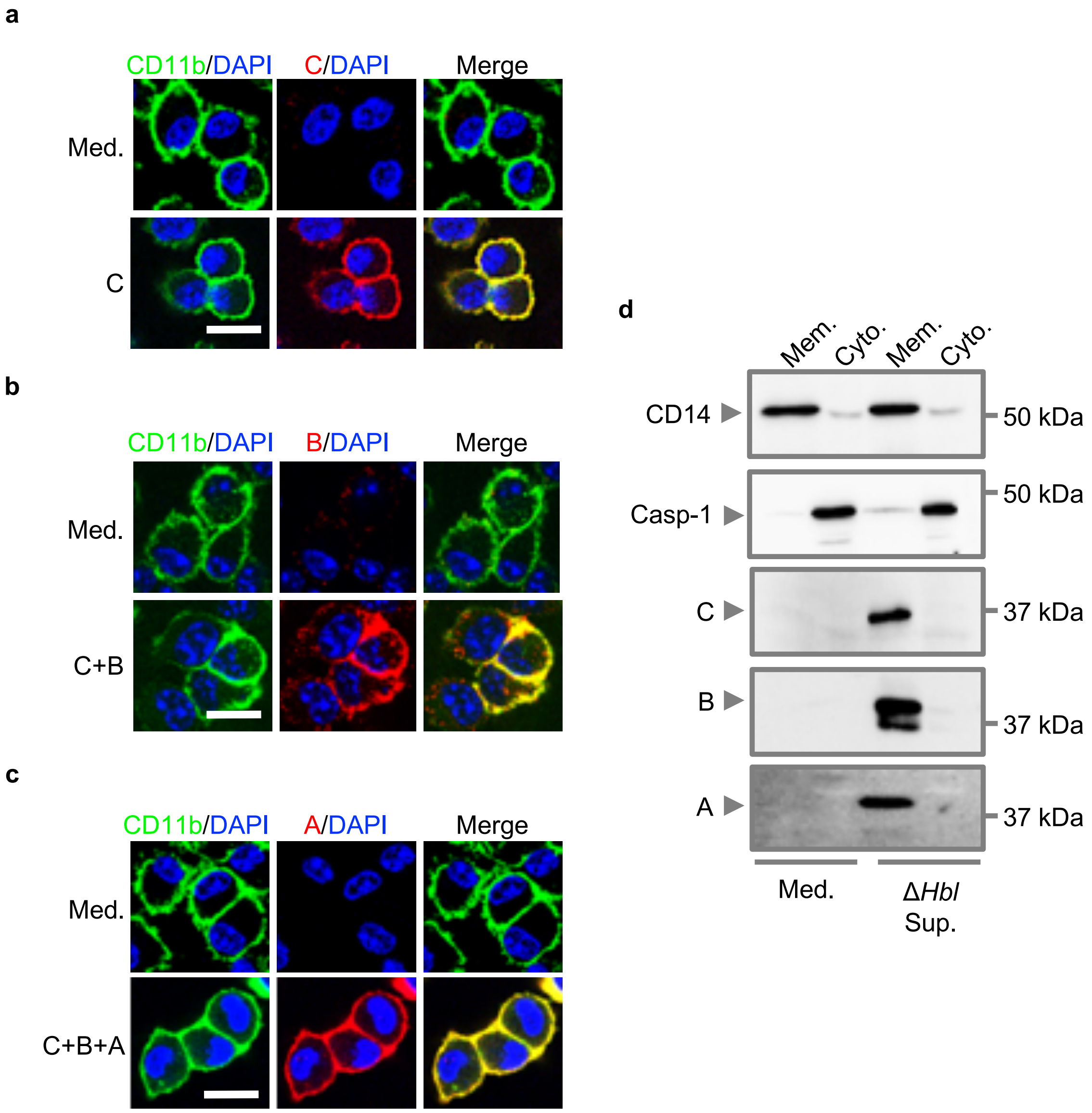
a
CD11b/DAPI
C/DAPI
Merge
Med.
C
b
CD11b/DAPI
B/DAPI
Merge
Med.
C+B
c
CD11b/DAPI
A/DAPI
Merge
Med.
C+B+A
d
Mem.
Cyto.
Mem.
Cyto.
CD14
50 kDa
Casp-1
50 kDa
C
37 kDa
B
37 kDa
A
37 kDa
Med.
ΔHbl
Sup.

**Supplementary Fig. 4 | NHE colocalises with and is present in the membrane fraction of BMDMs. a,** Immunofluorescent analysis of CD11b (green) and NHE-C (red) in WT BMDMs left untreated (Med.) or LPS-primed and assessed 1 h after treatment with NHE-C. **b,** Immunofluorescent analysis of CD11b (green) and NHE-B (red) in WT BMDMs left untreated or LPS-primed and assessed 1 h after treatment with NHE-C and NHE-B. **c,** Immunofluorescent analysis of CD11b (green) and NHE-A (red) in WT BMDMs left untreated or LPS-primed and assessed 30 min after treatment with NHE-C, NHE-B and NHE-A. **d**, Immunoblot analysis of CD14, caspase-1, C, B and A of WT BMDMs left untreated or assessed 1 h after treatment with the supernatant of Δ*Hbl B. cereus*. Mem, membrane fraction; Cyto, cytosolic fraction. Scale bars, 12 μm (**a-c**). Data are representative of three independent experiments (*n* = 3 in **a-d**). Source data are provided as a Source Data file.

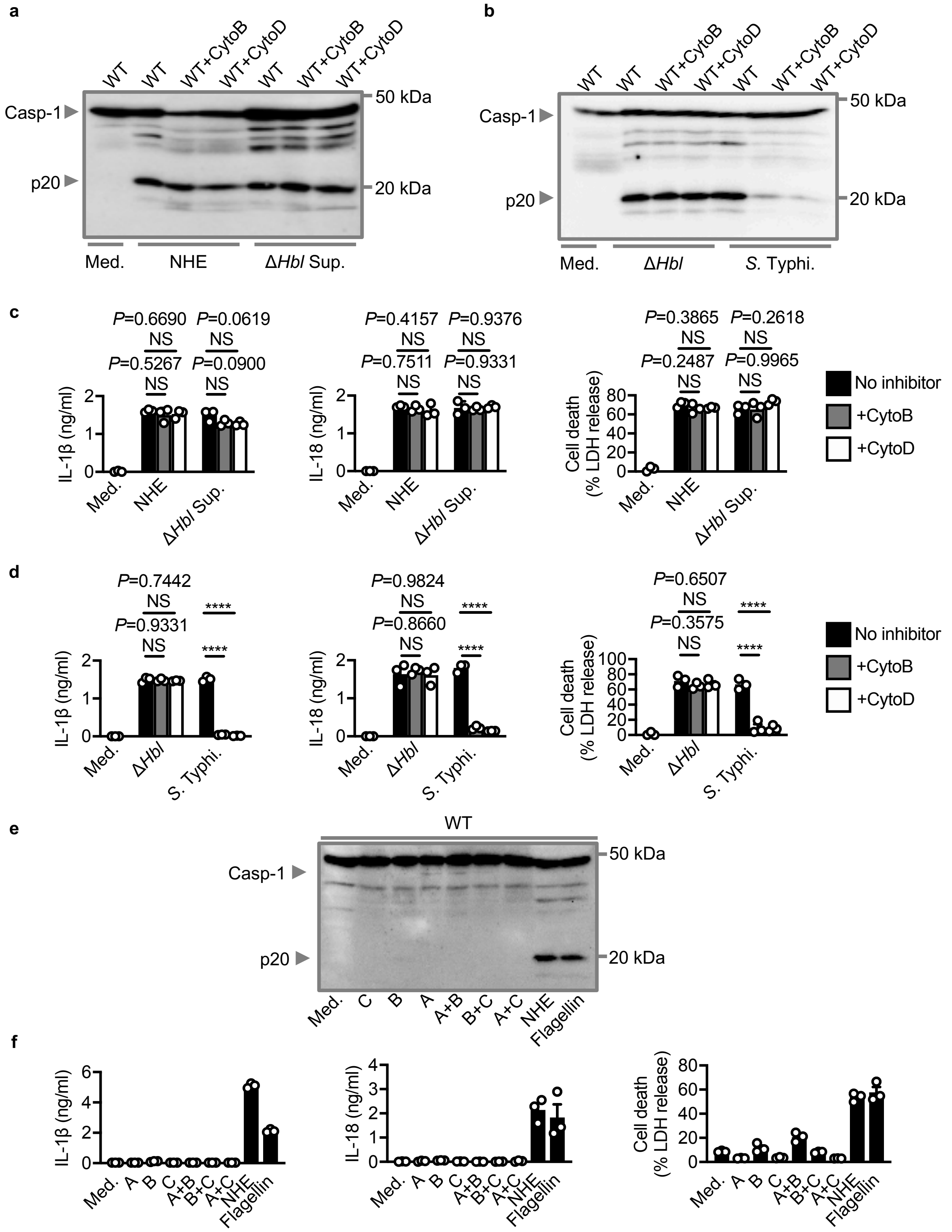

a
WT
WT
WT+CytoB
WT+CytoD
WT
WT+CytoB
WT+CytoD
Casp-1
p20
50 kDa
20 kDa
Med.
NHE
ΔHbl Sup.
b
WT
WT
WT+CytoB
WT+CytoD
WT
WT+CytoB
WT+CytoD
Casp-1
p20
50 kDa
20 kDa
Med.
ΔHbl
S. Typhi.
c
P=0.6690
NS
P=0.0619
NS
P=0.5267
NS
P=0.0900
NS
IL-1β (ng/ml)
Med.
NHE
ΔHbl Sup.
P=0.4157
NS
P=0.9376
NS
P=0.7511
NS
P=0.9331
NS
IL-18 (ng/ml)
Med.
NHE
ΔHbl Sup.
P=0.3865
NS
P=0.2618
NS
P=0.2487
NS
P=0.9965
NS
Cell death (% LDH release)
Med.
NHE
ΔHbl Sup.
No inhibitor
+CytoB
+CytoD
d
P=0.7442
NS
****
P=0.9331
NS
****
IL-1β (ng/ml)
Med.
ΔHbl
S. Typhi.
P=0.9824
NS
****
P=0.8660
NS
****
IL-18 (ng/ml)
Med.
ΔHbl
S. Typhi.
P=0.6507
NS
****
P=0.3575
NS
****
Cell death (% LDH release)
Med.
ΔHbl
S. Typhi.
No inhibitor
+CytoB
+CytoD
e
WT
Casp-1
p20
50 kDa
20 kDa
Med.
C
B
A
A+B
B+C
A+C
NHE
Flagellin
f
IL-1β (ng/ml)
IL-18 (ng/ml)
Cell death (% LDH release)
Med.
A
B
C
A+B
B+C
A+C
NHE
Flagellin

**Supplementary Fig. 5 | Cytosolic entry of NHE is not required for inflammasome activation. a,** Immunoblot analysis of caspase-1 of WT BMDMs left untreated or LPS-primed and assessed 3 h after treatment with NHE or the supernatant of Δ*Hbl B. cereus*, in the absence or presence of cytochalasin B (CytoB; 50 μM) or cytochalasin D (CytoD; 50 μM). **b,** Immunoblot analysis of caspase-1 of WT BMDMs left untreated or assessed 20 h after infection with Δ*Hbl B. cereus* (m.o.i. of 5) or *S.* Typhimurium (m.o.i. of 2), in the absence or presence of cytochalasin B (CytoB; 50 μM) or cytochalasin D (CytoD; 50 μM). **c,** Release of IL-1β and IL-18, and cell death of BMDMs as treated in **a**. **d,** Release of IL-1β and IL-18, and cell death of BMDMs as treated in **b**. **e,** Immunoblot analysis of caspase-1 of WT BMDMs left untreated or LPS-primed and assessed 3 h after transfection with one or two components of NHE, or after stimulation with NHE, or 5 h after transfection with flagellin of *S.* Typhimurium. **f,** Release of IL-1β and IL-18, and cell death of BMDMs as treated in **e**. Each symbol represents an independent experiment (**c**, **d** and **f**). NS, not statistically significant. ****$P$ < 0.0001 [one-way ANOVA with Dunnett's multiple-comparisons test (**c, d**)]. Data are representative of three independent experiments ($n$ = 3 in **a-f**; mean and s.e.m. in **c**, **d** and **f**). Source data are provided as a Source Data file.

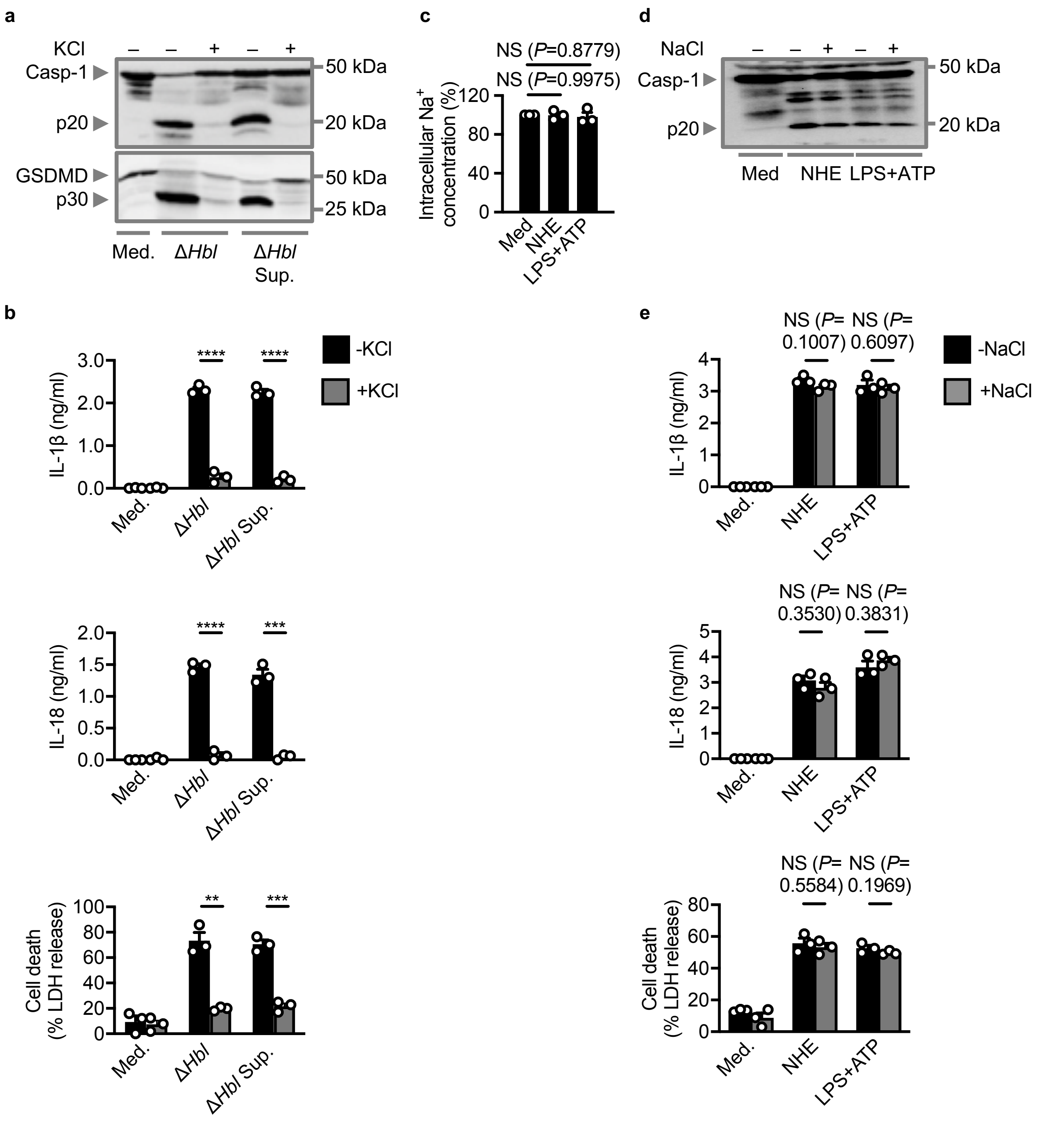
a
KCl – – + – +
Casp-1
p20
GSDMD
p30
50 kDa
20 kDa
50 kDa
25 kDa
Med.
ΔHbl
ΔHbl Sup.
c
NS (P=0.8779)
NS (P=0.9975)
Intracellular Na+ concentration (%)
Med
NHE
LPS+ATP
d
NaCl – – + – +
Casp-1
p20
50 kDa
20 kDa
Med
NHE
LPS+ATP
b
-KCl
+KCl
IL-1β (ng/ml)
IL-18 (ng/ml)
Cell death (% LDH release)
e
-NaCl
+NaCl
NS (P= 0.1007)
NS (P= 0.6097)
NS (P= 0.3530)
NS (P= 0.3831)
NS (P= 0.5584)
NS (P= 0.1969)

**Supplementary Fig. 6 | $Na^+$ flux is not required for NLRP3 inflammasome activation.**

**a**, Immunoblot analysis of caspase-1 and gasdermin D of WT BMDMs left untreated (Med.) or LPS-primed and assessed 20 h after infection with Δ*Hbl B. cereus* (m.o.i. of 5) or the supernatant of Δ*Hbl B. cereus* in the absence (-) or presence (+; 50 mM) of extracellular KCl; **b**, Release of IL-1β and IL-18, and death of WT BMDMs as in **a**. **c**, Inductively coupled plasma-optical emission spectrometry analysis of intracellular concentrations of $Na^+$ of BMDMs left untreated or assessed 2 h after treatment with NHE, or 30 mins after treatment with LPS+ATP. **d**, Immunoblot analysis of caspase-1 of WT BMDMs left untreated or LPS-primed and assessed 2 h after treatment with NHE, or 30 mins after treatment with LPS+ATP in the absence (-) or presence (+; 50 mM) of extracellular NaCl; **e**, Release of IL-1β and IL-18, and death of WT BMDMs as in **d**. Each symbol represents an independent experiment (**b, c** and **e**). NS, not statistically significant. $^{**}P < 0.01$ and $^{***}P < 0.001$, $^{****}P < 0.0001$ [student's unpaired *t*-test (**b** and **e**) or one-way ANOVA with Dunnett's multiple-comparisons test (**c**)]. Data are representative of three independent experiments ($n = 3$ in **a-e**; mean and s.e.m. in **b, c** and **e**). Source data are provided as a Source Data file.

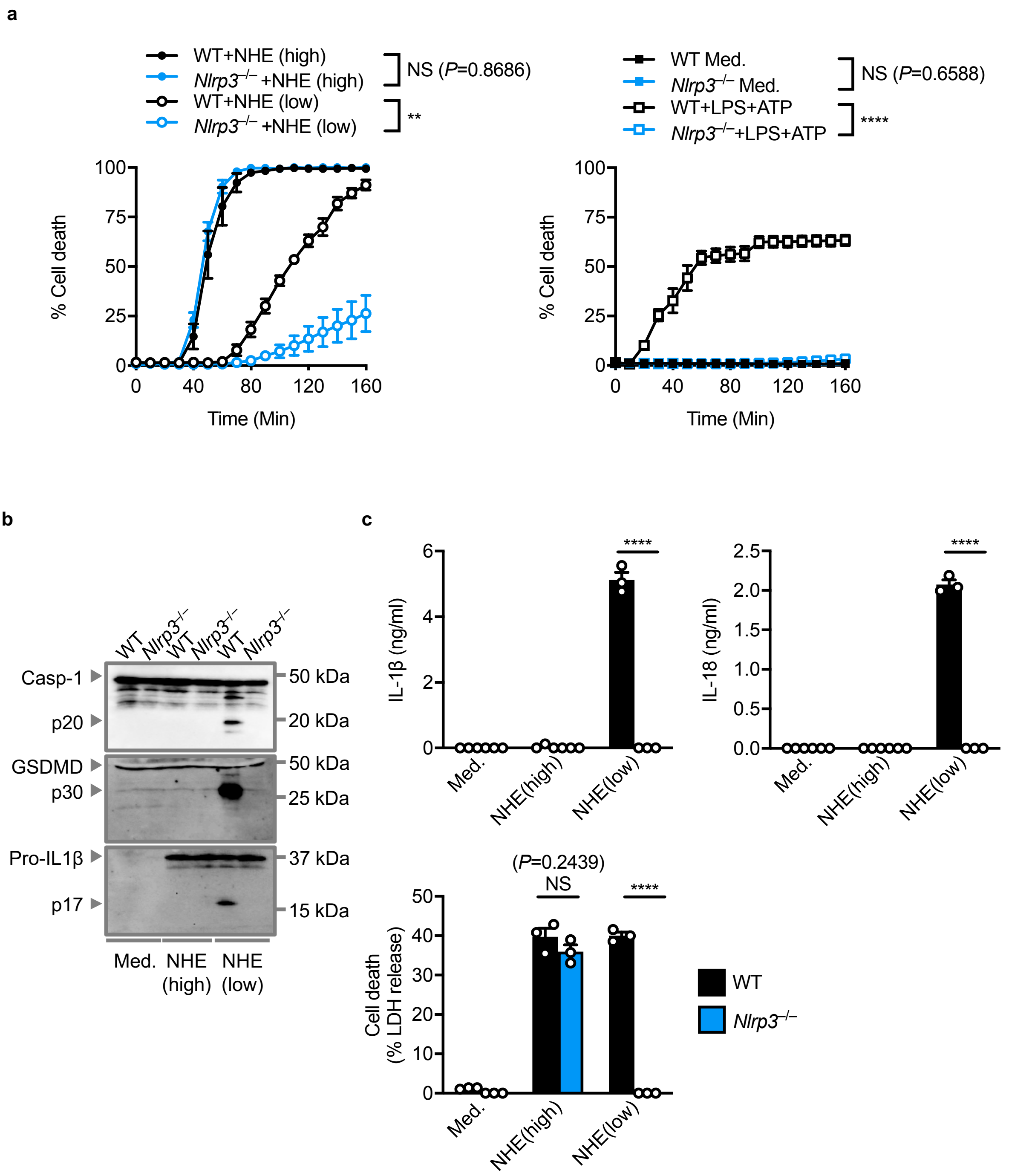
a
WT+NHE (high)
Nlrp3−/− +NHE (high)
WT+NHE (low)
Nlrp3−/− +NHE (low)
NS (P=0.8686)
**
% Cell death
Time (Min)
WT Med.
Nlrp3−/− Med.
WT+LPS+ATP
Nlrp3−/−+LPS+ATP
NS (P=0.6588)
****
b
WT
Nlrp3−/−
Casp-1
p20
GSDMD
p30
Pro-IL1β
p17
50 kDa
20 kDa
50 kDa
25 kDa
37 kDa
15 kDa
Med.
NHE (high)
NHE (low)
c
IL-1β (ng/ml)
IL-18 (ng/ml)
Med.
NHE(high)
NHE(low)
(P=0.2439)
NS
Cell death (% LDH release)
WT
Nlrp3−/−

**Supplementary Fig. 7 │ Bioavailability of NHE dictates cell death and activation of the NLRP3 inflammasome.** **a**, The viability of WT or *Nlrp3*$^{-/-}$ BMDMs left untreated (Med.) or after stimulation with 0.5 μM NHE (high) or 100 nM NHE (low) or with LPS+ATP, as determined by the IncuCyte live-imaging system. **b,** Immunoblot analysis of caspase-1 (top), gasdermin D (middle) and IL-1β (bottom) of WT or *Nlrp3*$^{-/-}$ BMDMs left untreated or LPS-primed and assessed 3 h after stimulation with 0.5 μM NHE or 100 nM NHE. **c,** Release of IL- 1β and IL-18, and death of WT BMDMs after treatment as in **b**. Each symbol represents an independent experiment **c**. NS, not statistically significant. $^{**}P < 0.01$ and $^{****}P < 0.0001$ [Student's unpaired *t*-test (**a** and **c**)]. Data are representative of three independent experiments ($n = 3$ in **a-c**; mean and s.e.m. in **a** and **c**). Source data are provided as a Source Data file.

**a**

Protein NHE-C sequence

**MQKRFYKKCLLTLMIAGVATSNAFPLHTFA**AEQNVKIQQENANDYSLGPAGFQDVMAQTTSSIFAMDSYAKLIQN
QQETDLSKISSINGELKGNMIQHQRDAKMNAAYWLNSMKPQIMKTDQNIINYNNTFQSYYNDMLIAIDQKDSGKL
KADLEKLYADIVKNQNEVDGLLGNLKAFRDRMAKDTNSFKEDTNQLTAILASTNAGIPALEQQINTYNDSIKKSN
DM**VIAGGVLCVALITCLAGGPMIAV**AKKDIANAEREIANLKDRISGAQAEVAILTDVKNKTTNMTETIDAAITAL
QNISNQWYTVGAKYNNLLQNVKGITPEEFTFIKEDLHTAKDSWKDVKDYTEKLHEGVAK

**b**

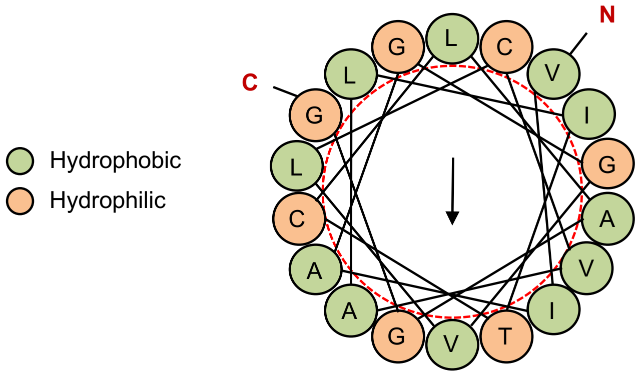


**c**

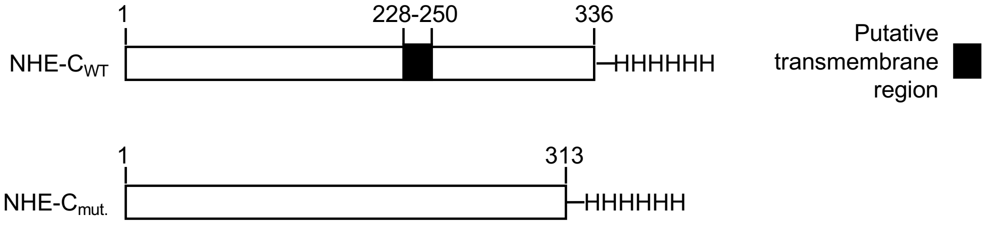


Protein NHE-C$_{mut.}$ sequence

MAEQNVKIQQENANDYSLGPAGFQDVMAQTTSSIFAMDSYAKLIQNQQETDLSKISSINGELKGNMIQHQRDAKM
NAAYWLNSMKPQIMKTDQNIINYNNTFQSYYNDMLIAIDQKDSGKLKADLEKLYADIVKNQNEVDGLLGNLKAFR
DRMAKDTNSFKEDTNQLTAILASTNAGIPALEQQINTYNDSIKKSNDMAKKDIANAEREIANLKDRISGAQAEVA
ILTDVKNKTTNMTETIDAAITALQNISNQWYTVGAKYNNLLQNVKGITPEEFTFIKEDLHTAKDSWKDVKDYTEK
LHEGVAK

**Supplementary Fig. 8 | The putative transmembrane region of NHE.** (**a**) Full-length amino acid sequence of NHE-C, highlighting the putative signal-peptide sequence (blue) and putative transmembrane region (red) based on bioinformatic-assisted analysis using the Membrane Protein Explorer (MPEx) Application. **b**, A standard helical wheel diagram showing the distribution of hydrophobic versus hydrophilic residues in the putative transmembrane region of the NHE-C, drawn using the HELIQUEST server. Arrow in the helical wheel represents the direction of the hydrophobic moment. **c**, Schematic of the sequences of NHE-$C_{WT}$ and NHE-$C_{mut.}$ (top). Amino acid sequence of NHE-$C_{mut.}$ (bottom).

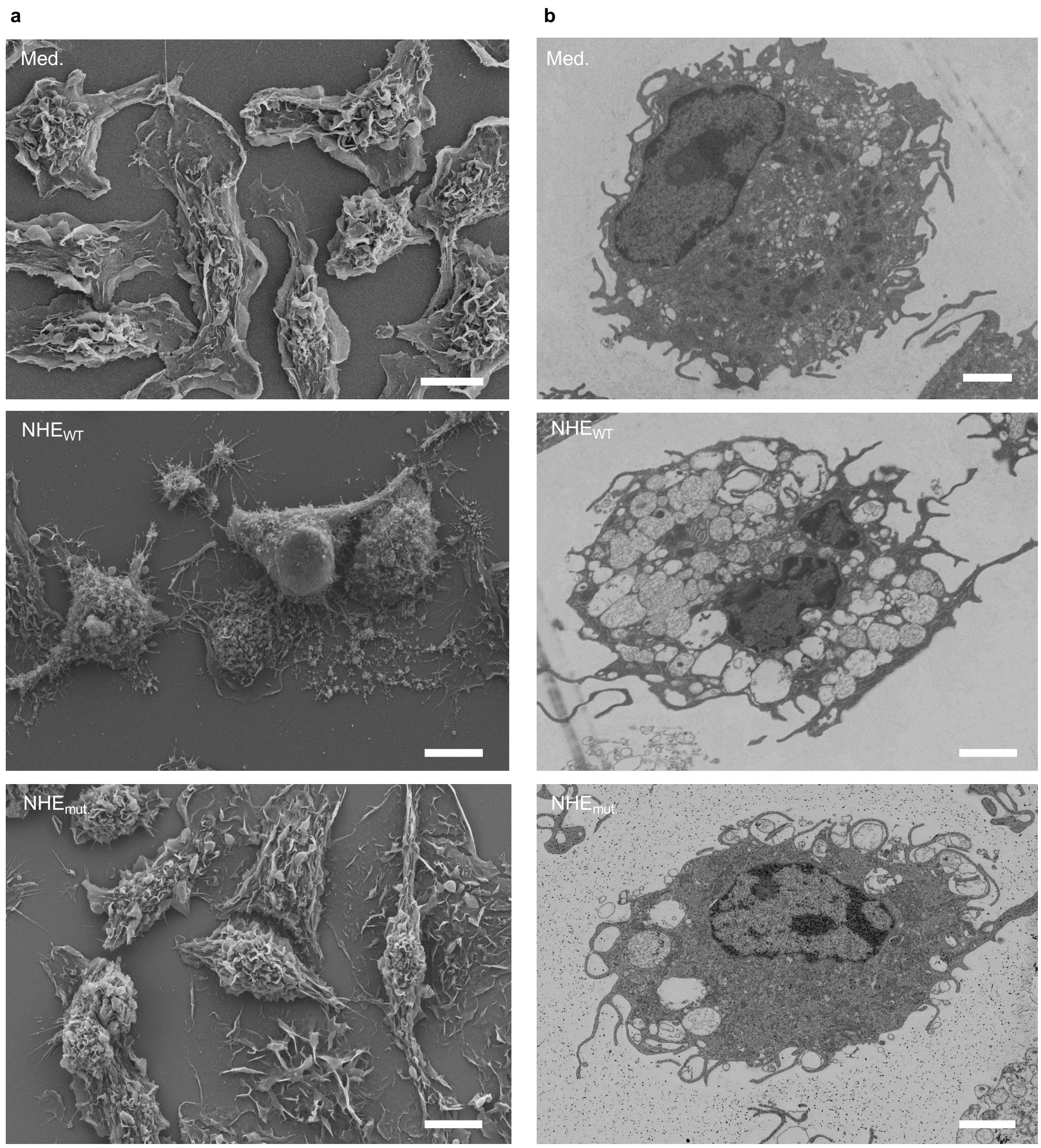

a
b
Med.
NHE$_{WT}$
NHE$_{mut.}$
Med.
NHE$_{WT}$
NHE$_{mut.}$

**Supplementary Fig. 9 | The putative transmembrane region of NHE facilitates the induction of cell death. a**, Overview of scanning electron microscopy analysis of WT BMDMs left untreated (Med., top left) or LPS-primed and assessed 2 h after stimulation with $NHE_{WT}$ (middle left) and $NHE_{mut.}$ (bottom left). **b**, Transmission electron microscopy analysis of WT BMDMs left untreated (top right) or LPS-primed and assessed 2 h after stimulation with $NHE_{WT}$ (middle right) and $NHE_{mut.}$ (bottom right). Scale bar, 10 µm (**a**), 2 µm (**b**). Data are representative of three independent experiments (*n* = 3 in **a, b**).

**a**

Protein HBL-B sequence

MGSEIEQTNNEDTALSANEVRMKETLQKAGLFAKSMNAYSYMLIKNPDVNFEGITINGYVDLPGRIVQDQKNARA
HAVTWDTKVKKQLLDTLNGIVEYDTTFDNYYETMIEAINTGDGETLKEGITDLRGEIQQNQKYAQQLIEELTKLR
DSIGHDVRAFGSNKELLQSILKNQGADVDADQKRLEEVLGSVNYYKQLESDG**FNVMKGAILGLPIIGGIIVGVA**R
DNLGKLEPLLAELRQTVDYKVTLNRVVGVAYSNINEMHKALDDAINALTYMSTQWHDLDSQYSGVLGHIENAAQK
ADQNKFKFLKPNLNAAKDSWKTLRTDAVTLKEGIKELKVETVTPQK

Protein HBL-$B_{mut.}$ sequence

MGSEIEQTNNEDTALSANEVRMKETLQKAGLFAKSMNAYSYMLIKNPDVNFEGITINGYVDLPGRIVQDQKNARA
HAVTWDTKVKKQLLDTLNGIVEYDTTFDNYYETMIEAINTGDGETLKEGITDLRGEIQQNQKYAQQLIEELTKLR
DSIGHDVRAFGSNKELLQSILKNQGADVDADQKRLEEVLGSVNYYKQLESDGGVARDNLGKLEPLLAELRQTVDY
KVTLNRVVGVAYSNINEMHKALDDAINALTYMSTQWHDLDSQYSGVLGHIENAAQKADQNKFKFLKPNLNAAKDS
WKTLRTDAVTLKEGIKELKVETVTPQK

**b**

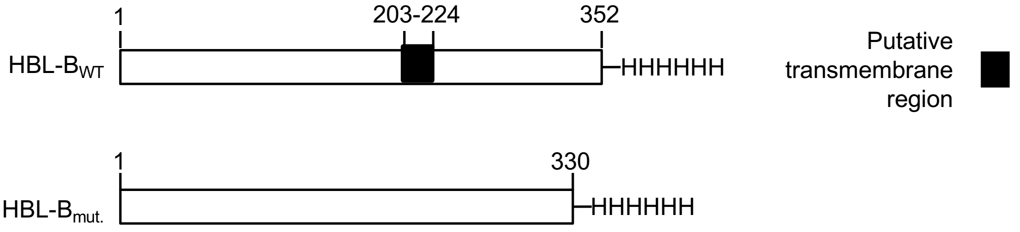


**c**

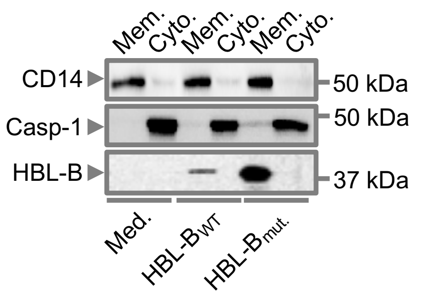


**d**

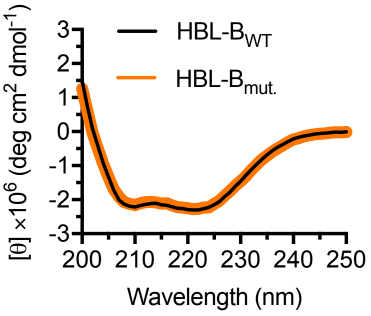

**Supplementary Fig. 10 | The putative transmembrane region in HBL-B is not required for membrane binding.** **a**, Full-length amino acid sequences of HBL-B (top) and HBL-$B_{mut.}$ (below), highlighting the putative transmembrane region (red) based on bioinformatic-assisted analysis using the Membrane Protein Explorer (MPEx) Application. **b**, Schematic of the sequences of HBL-$B_{WT}$ and HBL-$B_{mut.}$ **c**, Immunoblot analysis of CD14, caspase-1, and HBL-B of WT BMDMs left untreated (Med.) or assessed 1 h after treatment with HBL-$B_{WT}$ or HBL-$B_{mut.}$. Mem, membrane fraction; Cyto, cytosolic fraction. **d**, Circular dichroism analysis of the secondary structures of HBL-$B_{WT}$ or HBL-$B_{mut.}$. Data are representative of three independent experiments (*n* = 3 in **c**; *n* = 1 in **d**). Source data are provided as a Source Data file.

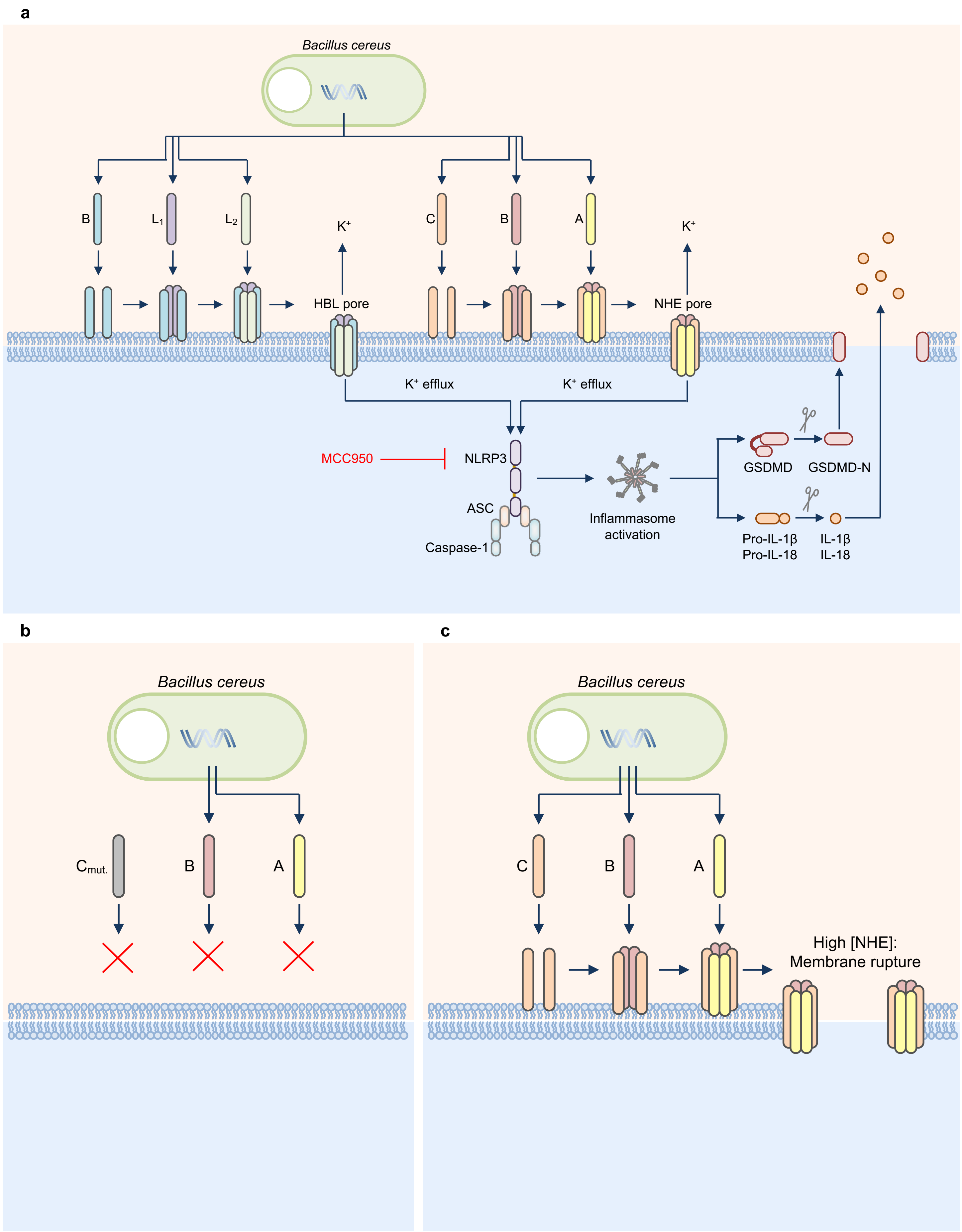
a
Bacillus cereus
B
L1
L2
K+
HBL pore
C
B
A
K+
NHE pore
K+ efflux
K+ efflux
MCC950
NLRP3
ASC
Caspase-1
Inflammasome activation
GSDMD
GSDMD-N
Pro-IL-1β
Pro-IL-18
IL-1β
IL-18
b
Bacillus cereus
Cmut.
B
A
c
Bacillus cereus
C
B
A
High [NHE]:
Membrane rupture

**Supplementary Fig. 11 │ Model of *B. cereus*-induced activation of the NLRP3 inflammasome.** **a**, Model depicting the conserved mechanism of action of HBL and NHE, exploited for sensing by the NLRP3 inflammasome. **b**, Model showing the necessity of the putative transmembrane domain in NHE-C for driving assembly of the tripartite NHE toxin to the plasma membrane of BMDMs. **c**, Model depicting cellular lysis and membrane rupture of BMDMs after treatment with high concentrations of NHE.

## SUPPLEMENTARY TABLES

**Supplementary Table 1 | *B. cereus* strains used in this study and their sources.**

| Strain ID | Source | Reference |
|---|---|---|
| ATCC 14579 | Air | ATCC |
| ATCC 10876 | Contaminated Flask | ATCC |
| Δ*Hbl* | ATCC 10876 | [1] |
| Δ*Nhe* | F837/76 | [2,3] |

**Supplementary Table 2 | List of RT-PCR primers used in this study.**

| Target gene | Primer name | Primer sequence | Reference |
|---|---|---|---|
| *Gapdh* | Gapdh-F | 5'-CGT CCC GTA GAC AAA ATG GT-3' | [4] |
| | Gapdh-R | 5'-TTG ATG GCA ACA ATC TCC AC-3' | |
| *Cxcl1* | mKc-F | 5'-CAA TGA GCT GCG CTG TCA GTG-3' | [5] |
| | mKc-R | 5'-CTT GGG GAC ACC TTT TAG CAT C-3' | |
| *Il1b* | mIl1b-F | 5'-GAC CTT CCA GGA TGA GGA CA-3' | [4] |
| | mIl1b-R | 5'-AGC TCA TAT GGG TCC GAC AG-3' | |
| *Tnf* | mTnf-F | 5'-CAT CTT CTC AAA ATT CGA GTG ACA A-3' | [4] |
| | mTnf-R | 5'-TGG GAG TAG ACA AGG TAC AAC CC-3' | |

## SUPPLEMENTARY REFERENCES


1. Sastalla, I., *et al.* The *Bacillus cereus* Hbl and Nhe tripartite enterotoxin components assemble sequentially on the surface of target cells and are not interchangeable. *PLoS One* **8**, e76955 (2013).
2. Tausch, F., *et al.* Evidence for Complex Formation of the *Bacillus cereus* Haemolysin BL Components in Solution. *Toxins (Basel)* **9**(2017).
3. Turnbull, P.C., Nottingham, J.F. & Ghosh, A.C. A severe necrotic enterotoxin produced by certain food, food poisoning and other clinical isolates of *Bacillus cereus*. *Br J Exp Pathol* **58**, 273-280 (1977).
4. Man, S.M., *et al.* IRGB10 Liberates Bacterial Ligands for Sensing by the AIM2 and Caspase-11-NLRP3 Inflammasomes. *Cell* **167**, 382-396.e317 (2016).
5. Man, S.M., *et al.* Critical Role for the DNA Sensor AIM2 in Stem Cell Proliferation and Cancer. *Cell* **162**, 45-58 (2015).
