# Supplementary material for: Bacillus cereus non-haemolytic enterotoxin activates the NLRP3 inflammasome
Source: Nat Commun. 2020 Feb 6;11:760. doi: 10.1038/s41467-020-14534-3 (PMC7005308; doi:10.1038/s41467-020-14534-3)
Supplement: Supplementary file 2 — Reporting Summary [file 41467_2020_14534_MOESM2_ESM.pdf]

nature research

Corresponding author(s): Si Ming Man

Last updated by author(s): Dec 19, 2019

Reporting Summary

Nature Research wishes to improve the reproducibility of the work that we publish. This form provides structure for consistency and transparency in reporting. For further information on Nature Research policies, see Authors & Referees and the Editorial Policy Checklist .

Statistics

For all statistical analyses, confirm that the following items are present in the figure legend, table legend, main text, or Methods section.

n/a	Confirmed	
☐	☒	The exact sample size (n) for each experimental group/condition, given as a discrete number and unit of measurement
☐	☒	A statement on whether measurements were taken from distinct samples or whether the same sample was measured repeatedly
☐	☒	The statistical test(s) used AND whether they are one- or two-sided<br>*Only common tests should be described solely by name; describe more complex techniques in the Methods section.*
☒	☐	A description of all covariates tested
☐	☒	A description of any assumptions or corrections, such as tests of normality and adjustment for multiple comparisons
☐	☒	A full description of the statistical parameters including central tendency (e.g. means) or other basic estimates (e.g. regression coefficient) AND variation (e.g. standard deviation) or associated estimates of uncertainty (e.g. confidence intervals)
☐	☒	For null hypothesis testing, the test statistic (e.g. F, t, r) with confidence intervals, effect sizes, degrees of freedom and P value noted<br>*Give P values as exact values whenever suitable.*
☒	☐	For Bayesian analysis, information on the choice of priors and Markov chain Monte Carlo settings
☒	☐	For hierarchical and complex designs, identification of the appropriate level for tests and full reporting of outcomes
☒	☐	Estimates of effect sizes (e.g. Cohen's d, Pearson's r), indicating how they were calculated

Our web collection on statistics for biologists contains articles on many of the points above.

Software and code

Policy information about availability of computer code

Data collection	No software used.
Data analysis	GraphPad Prism 6.0 , LAS AF version 2.7.3., Image Lab Software 6.0, IncuCyte Zoom 2016A, MPEx server, HELIQUEST server and Syngistix.

For manuscripts utilizing custom algorithms or software that are central to the research but not yet described in published literature, software must be made available to editors/reviewers. We strongly encourage code deposition in a community repository (e.g. GitHub). See the Nature Research guidelines for submitting code & software for further information.

Data

Policy information about availability of data

All manuscripts must include a data availability statement. This statement should provide the following information, where applicable:

- Accession codes, unique identifiers, or web links for publicly available datasets
- A list of figures that have associated raw data
- A description of any restrictions on data availability

The data that support the findings of this study are included in the Source Data file, and are also available from the corresponding author upon request.

Field-specific reporting

Please select the one below that is the best fit for your research. If you are not sure, read the appropriate sections before making your selection.

☒ Life sciences ☐ Behavioural & social sciences ☐ Ecological, evolutionary & environmental sciences

For a reference copy of the document with all sections, see nature.com/documents/nr-reporting-summary-flat.pdf

Life sciences study design

All studies must disclose on these points even when the disclosure is negative.

Sample size	Sample sizes are large enough to detect the effects of interest holding biological significance.
Data exclusions	No data were excluded.
Replication	The experimental findings were reliably reproduced. See details in the figure legends and Source Data file.
Randomization	No randomization was performed.
Blinding	No blinding was performed.

Reporting for specific materials, systems and methods

We require information from authors about some types of materials, experimental systems and methods used in many studies. Here, indicate whether each material, system or method listed is relevant to your study. If you are not sure if a list item applies to your research, read the appropriate section before selecting a response.

Materials & experimental systems

n/a	Involved in the study	
☐	☒	Antibodies
☐	☒	Eukaryotic cell lines
☒	☐	Palaeontology
☐	☒	Animals and other organisms
☒	☐	Human research participants
☒	☐	Clinical data

Methods

n/a	Involved in the study	
☒	☐	ChIP-seq
☒	☐	Flow cytometry
☒	☐	MRI-based neuroimaging

Antibodies

Antibodies used	All antibodies used are described in the manuscript.
Validation	All primary antibodies were validated.

Eukaryotic cell lines

Policy information about cell lines

Cell line source(s)	L-929, RMA-S and Vero cell lines were kindly provided by Prof. David Tscharke.<br>MutuDCs were a kind gift from Prof. Hans Acha-Orbea and Dr Justine Mintern.<br>HEK293T, Jurkat, K-562 and U266B1 cell lines were a kind gift from Prof. Carola Vinuesa.<br>CaCo-2, HT-29 and LS 174T cell lines were a kind gift from Dr. Nadeem Kaakoush.<br>CT26 and MC38 cell lines were a kind gift from Prof. Chris Parish.<br><br>MDCK from https://www.atcc.org/products/all/CCL-34.aspx<br>THP-1 from https://www.atcc.org/products/all/TIB-202.aspx
Authentication	Cell lines were verified by manufacturer's website and Identity of these cell lines were frequently checked by their morphological features.<br><br>MDCK from https://www.atcc.org/products/all/CCL-34.aspx<br>THP-1 from https://www.atcc.org/products/all/TIB-202.aspx
Mycoplasma contamination	All cell lines were tested to be mycoplasma-negative by laboratories of origin or manufacturer.
Commonly misidentified lines (See ICLAC register)	No commonly misidentified cell lines are used in this study.

Animals and other organisms

Policy information about studies involving animals; ARRIVE guidelines recommended for reporting animal research

Laboratory animals	C57BL/6 mice, both males and females between 6-8 weeks old.
Wild animals	N/A
Field-collected samples	N/A
Ethics oversight	Mice were bred and maintained at The Australian National University and all animal studies were conducted in accordance with the Protocol Number A2017/05 approved by The Australian National University Animal Experimentation Ethics Committee.

Note that full information on the approval of the study protocol must also be provided in the manuscript.
